# In‐depth proteomic analysis reveals unique subtype‐specific signatures in human small‐cell lung cancer

**DOI:** 10.1002/ctm2.1060

**Published:** 2022-09-23

**Authors:** Beáta Szeitz, Zsolt Megyesfalvi, Nicole Woldmar, Zsuzsanna Valkó, Anna Schwendenwein, Nándor Bárány, Sándor Paku, Viktória László, Helga Kiss, Edina Bugyik, Christian Lang, Attila Marcell Szász, Luciana Pizzatti, Krisztina Bogos, Mir Alireza Hoda, Konrad Hoetzenecker, György Marko‐Varga, Peter Horvatovich, Balázs Döme, Karin Schelch, Melinda Rezeli

**Affiliations:** ^1^ Division of Oncology, Department of Internal Medicine and Oncology Semmelweis University Budapest Hungary; ^2^ National Korányi Institute of Pulmonology Budapest Hungary; ^3^ Department of Thoracic Surgery Medical University of Vienna Vienna Austria; ^4^ Department of Thoracic Surgery, National Institute of Oncology Semmelweis University Budapest Hungary; ^5^ Division of Clinical Protein Science & Imaging, Department of Clinical Sciences (Lund) and Department of Biomedical Engineering Lund University Lund Sweden; ^6^ Laboratory of Molecular Biology and Proteomics of Blood/LADETEC, Institute of Chemistry Federal University of Rio de Janeiro Rio de Janeiro Brazil; ^7^ First Department of Pathology and Experimental Cancer Research Semmelweis University Budapest Hungary; ^8^ University of Pécs Pécs Hungary; ^9^ Department of Bioinformatics Semmelweis University Budapest Hungary; ^10^ Department of Analytical Biochemistry, Groningen Research Institute of Pharmacy University of Groningen Groningen The Netherlands; ^11^ Department of Translational Medicine Lund University Lund Sweden; ^12^ Center for Cancer Research Medical University of Vienna Vienna Austria

**Keywords:** diagnostic biomarkers, molecular targets, proteomics, secretome, small‐cell lung cancer, subtype, transcriptomics

## Abstract

**Background:**

Small‐cell lung cancer (SCLC) molecular subtypes have been primarily characterized based on the expression pattern of the following key transcription regulators: *ASCL1* (SCLC‐A), *NEUROD1* (SCLC‐N), *POU2F3* (SCLC‐P) and *YAP1* (SCLC‐Y). Here, we investigated the proteomic landscape of these molecular subsets with the aim to identify novel subtype‐specific proteins of diagnostic and therapeutic relevance.

**Methods:**

Pellets and cell media of 26 human SCLC cell lines were subjected to label‐free shotgun proteomics for large‐scale protein identification and quantitation, followed by in‐depth bioinformatic analyses. Proteomic data were correlated with the cell lines’ phenotypic characteristics and with public transcriptomic data of SCLC cell lines and tissues.

**Results:**

Our quantitative proteomic data highlighted that four molecular subtypes are clearly distinguishable at the protein level. The cell lines exhibited diverse neuroendocrine and epithelial–mesenchymal characteristics that varied by subtype. A total of 367 proteins were identified in the cell pellet and 34 in the culture media that showed significant up‐ or downregulation in one subtype, including known druggable proteins and potential blood‐based markers. Pathway enrichment analysis and parallel investigation of transcriptomics from SCLC cell lines outlined unique signatures for each subtype, such as upregulated oxidative phosphorylation in SCLC‐A, DNA replication in SCLC‐N, neurotrophin signalling in SCLC‐P and epithelial–mesenchymal transition in SCLC‐Y. Importantly, we identified the *YAP1*‐driven subtype as the most distinct SCLC subgroup. Using sparse partial least squares discriminant analysis, we identified proteins that clearly distinguish four SCLC subtypes based on their expression pattern, including potential diagnostic markers for SCLC‐Y (e.g. GPX8, PKD2 and UFO).

**Conclusions:**

We report for the first time, the protein expression differences among SCLC subtypes. By shedding light on potential subtype‐specific therapeutic vulnerabilities and diagnostic biomarkers, our results may contribute to a better understanding of SCLC biology and the development of novel therapies.

## Graphical abstract

1. The first mass spectrometry–based proteomic analysis on small‐cell lung cancer (SCLC) cell lines, reporting unique proteomic profiles of its proposed molecular subtypes.
2. SCLC subgroups detected by proteomics are consistent with mRNA‐based subtypes.
3. Unique proteomic profiles of SCLC subtypes highlight potential subtype‐specific therapeutic vulnerabilities and diagnostic biomarkers.

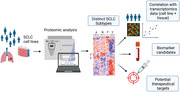

## 1 INTRODUCTION

Small‐cell lung cancer (SCLC) represents about 13%–15% of all lung cancers and with a 5‐year survival rate of less than 7%, it remains one of the most lethal forms of malignant diseases.^1^, ^2^, ^3^ It has a very aggressive course and is characterized by extensive chromosomal rearrangements, high mutation burden and almost universal inactivation of the tumour suppressor genes *TP53* and *RB1*.^1^, ^4^ Therefore, the vast majority of SCLC patients are diagnosed with extensive‐stage disease when surgery is not feasible, and the treatment options are mostly limited to cytotoxic chemotherapy and radiation.^4^ Importantly, targeted therapies for these patients have so far failed, and the success of immunotherapy in non‐SCLC has not been reflected in SCLC.^3^, ^5^

Although SCLC has been formerly considered a homogeneous disease with a single morphological type, recent advances in SCLC research have led to the development of subtype‐specific classifications primarily based on neuroendocrine (NE) features and unique molecular profiles.^3^, ^6^, ^7^ Accordingly, SCLC can be classified into NE‐high and NE‐low subtypes based on the expression pattern of key NE markers (i.e. *SYP*, *CHGA*, *NCAM1/CD56* and *GRP*).^1^, ^8^ To further elucidate the clinicopathological relevance of NE subtypes, Zhang et al. developed a numeric score to evaluate the degree of NE differentiation in SCLC.^7^ By using the top 50 genes most strongly associated with NE differentiation, they successfully separated NE high and low subsets of SCLC tumours and cell lines and showed that these subtypes had widely different growth properties and morphology.^7^ Of note, some SCLCs lack NE differentiation and are termed non‐NE tumours.^6^ Importantly, the NE differentiation also serves as a framework for the recently emerged molecular subtypes.^6^ These newly proposed molecular subgroups (SCLC‐A, SCLC‐N, SCLC‐P and SCLC‐Y) have been defined based on the expression pattern of transcription factors *ASCL1*, *NEUROD1*, *POU2F3* and *YAP1*, respectively.^4^, ^6^ SCLC‐P and SCLC‐Y show a non‐NE phenotype, whereas SCLC‐A and SCLC‐N are classified as NE tumours (NE high and low, respectively).^4^, ^6^ Moreover, our group also found recently that high POU2F3 expression is associated with improved survival outcomes, whereas elevated ASCL1 expression is an independent negative prognosticator in surgically treated SCLC patients,^9^ highlighting the prognostic relevance of SCLC subgroups. So far, the vast majority of SCLC profiling studies have been conducted by analysing transcriptomic data and gene expression profiles of clinical samples and preclinical models, such as patient‐derived and circulating tumour cell‐derived SCLC xenografts.^6^, ^8^, ^10^, ^11^ Although recent immunohistochemistry (IHC) analyses confirmed that subtype‐specific markers are indeed detectable in human tumour tissue samples, these studies failed to distinguish a unique *YAP1*‐driven subtype.^12^ Of note, based on tumour expression data analysis by non‐negative matrix factorization, Gay et al. proposed a unique inflamed (SCLC‐I) subtype characterized by an inflamed gene signature as an alternative to the YAP1‐defined subtype.^13^ Additionally, not all previously expected RNA‐based correlation patterns between subtype markers and NE features could have been validated by IHC either.^12^ Therefore, subtype classification based on transcriptional profiling might not be exclusively representative concerning protein expression.

Mass spectrometry (MS)‐based proteomics enables a large‐scale analysis of complex biological systems, such as cells, tissues or blood plasma. Parallel detection and quantitation of thousands of proteins, including those with lower abundance, is feasible with modern high‐resolution mass spectrometers and advanced sample preparation workflows, thus achieving a better understanding of molecular interactions and signalling pathways in cancer.^14^, ^15^ The scarcity of available material has so far hindered a comprehensive proteomic examination of SCLC tissues, whereas the proteome of non‐SCLC tissues were thoroughly mapped previously.^16^, ^17^ To date, proteomic studies considered SCLC as a single entity and compared SCLC samples with normal bronchial epithelial tissues,^18^ non‐SCLC cell lines^19^ or carcinoid tumour tissues.^20^ Of note, these studies reported relatively low number of identifiable proteins, ranging from 193 to 1991. A proteomic study of 949 cancer cell lines from 28 tissue types, including 57 SCLC cell lines, was published only recently, in which a total of 8498 proteins were quantified.^21^

In the current work, we conducted a comparative proteomic analysis of SCLC subtypes using cell lines categorized based on the relative expression of four key transcription factors. Our label‐free shotgun proteomic approach assessing both the cell pellet (CP) and culture media (CM) resulted in the confident quantification of nearly 9000 proteins. This comprehensive proteomic evaluation of SCLC cell lines integrated with knowledge from existing transcriptomic datasets can give a clearer definition of SCLC subtypes and might provide insights into their specific features that govern therapy response.

## 2 MATERIALS AND METHODS

### 2.1 Cell culture

Human SCLC cell lines were maintained in RPMI‐1640 with 10% fetal calf serum (Sigma Chemical Co.), 100 U/ml penicillin and 10 mg/ml streptomycin (Sigma Chemical Co.) at 37°C in a humidified incubator with 5% CO_2_. SCLC cell lines were either purchased from ATCC or kindly provided by our collaborators. All cell lines were regularly checked for mycoplasma contamination using the luminescence‐based MycoAlert mycoplasma detection kit (Lonza) with supernatant from cells cultured for >3 days and used within 10 passages after authentication. The cell lines and their key characteristics are listed in Table 1.

**TABLE 1:** Cell lines included in the study, and their general characteristics

Cell line ID	Other cell line ID	Subtype	Cell line origin	Chemotherapy	Culture type
DMS153	CRL‐2064	SCLC‐A	Metastatic	Post‐chemo	Semi‐adherent
DMS53	CRL‐2062	SCLC‐A	Lung	Chemo‐naïve	Adherent
H146	HTB‐173	SCLC‐A	Metastatic	Chemo‐naïve	Suspension
H1688	CCL‐257	SCLC‐A	Metastatic	Chemo‐naïve	Adherent
H1882	CRL‐5903	SCLC‐A	Metastatic	N/A	Adherent
H209	HTB‐172	SCLC‐A	Metastatic	Chemo‐naïve	Suspension
H378	CRL‐5808	SCLC‐A	Lung	Post‐chemo	Suspension
SHP77	CRL‐2195	SCLC‐A	Lung	N/A	Adherent
GLC4	N/A	SCLC‐N	Pleural eff.	Chemo‐naïve	Suspension
H1694	CRL‐5888	SCLC‐N	Lung	N/A	Semi‐adherent
H2171	CRL‐5929	SCLC‐N	Pleural eff.	Post‐chemo	Suspension
H446	HTB‐171	SCLC‐N	Pleural eff.	N/A	Adherent
H524	CRL‐5831	SCLC‐N	Metastatic	Post‐chemo	Suspension
H82	HTB‐175	SCLC‐N	Metastatic	N/A	Semi‐adherent
N417	CRL‐5809	SCLC‐N	Lung	N/A	Suspension
COR‐L311	N/A	SCLC‐P	Lung	Post‐chemo	Suspension
H1048	CRL‐5853	SCLC‐P	Pleural eff.	N/A	Adherent
H211	CRL‐5824	SCLC‐P	Lung	Post‐chemo	Suspension
H526	CRL‐5811	SCLC‐P	Metastatic	Chemo‐naïve	Suspension
CRL‐2066	DMS 114	SCLC‐Y	Lung	Chemo‐naïve	Adherent
CRL‐2177	SW1271	SCLC‐Y	Lung	N/A	Adherent
H1341	CRL‐5864	SCLC‐Y	Metastatic	N/A	Adherent
H196	CRL‐5823	SCLC‐Y	Pleural eff.	Post‐chemo	Adherent
H372	N/A	SCLC‐Y	Metastatic	N/A	Adherent
H841	CRL‐5845	SCLC‐Y	Lung	Post‐chemo	Adherent
HLHE	N/A	SCLC‐Y	Metastatic	N/A	Adherent

*Note*: Pleural eff., pleural effusion.

Abbreviations: N/A, not available; SCLC, small‐cell lung cancer.

### 2.2 RNA isolation and qPCR

Cells were incubated in T25 flasks until 70% confluency. Total RNA was isolated with TRIzol reagent and reverse transcribed with MMLV reverse transcriptase (Thermo Fisher Scientific) according to the manufacturer's protocol. Gene expression was analysed by quantitative polymerase chain reaction (qPCR) using TaqMan gene expression assays on the Applied Biosystems 7500 Fast Real‐Time PCR System (Assay IDs: *ASCL1*: Hs00269932_m1, *NEUROD1*: Hs00159598_m1, *POU2F3*: Hs00205009_m1, *YAP1*: Hs00371735_m1, *GAPDH*: Hs02786624_g1; Thermo Fisher Scientific). Gene expression was calculated as 2^−Δ^ *^Ct^* using glyceraldehyde 3‐phosphate dehydrogenase (*GAPDH*) as reference gene.^22^

### 2.3 Sample processing for proteomics

CPs and CM from 26 cell lines were processed and subjected to an MS‐based proteomic analysis. In brief, the CPs were solubilized with protein extraction buffer (25 mM dithiothreitol [DTT], 10% sodium dodecyl sulphate (SDS), 100 mM triethylammonium bicarbonate [TEAB], pH 8), incubating for 5 min at 95°C with 500 rpm shaking. The volume of the buffer was adjusted to the number of cells in each sample, that is 250 μl of protein extraction buffer was added to samples containing 5 M cells. Proteins were extracted via a 20 min sonication at 4°C (Bioruptor Plus, Diagenode) with 40 cycles (15 s on/15 s off), followed by a brief centrifugation at 20000 × *g* at 18°C, discarding the cell debris. Protein determination was performed using a Pierce 660 nm Protein Assay kit (Thermo Scientific), following the manufacturer's instructions.

Additionally, the filtered CM samples were concentrated using spin concentrators (5K 4 ml, Agilent Technologies) to ∼100 μl. Protein determination was performed with NanoDrop (DeNovix DS‐11 FX +). Prior to digestion, SDS was added to a final concentration of 3%, and 100 mM TEAB was added to increase the pH. Reduction was performed with 10 mM DTT and a 1 h incubation at 37°C.

Protein digestion for both the CP and media (100 μg of proteins per sample) was accomplished using the S‐Trap technology (ProtiFi) with few modifications, as previously described by our group.^23^ In brief, the samples were alkylated with 50 mM iodoacetamide and acidified with 1.2% phosphoric acid (final concentration). Then, S‐Trap binding buffer was added (90% methanol, 100 mM TEAB) to 7× the final sample volume, and the samples were transferred to the S‐Trap 96‐well digestion plate. Captured proteins were washed four times with 200 μl S‐Trap binding buffer and brief centrifugations (2 min at 1000 × *g*). Digestion buffer (50 mM TEAB) containing LysC at 1:50 enzyme‐to‐protein ratio was added on top of the filters, incubating for 2 h at 37°C. Next, digestion buffer containing trypsin at 1:50 enzyme‐to‐protein ratio was added to the samples, incubating overnight at 37°C. On the following day, the peptides were eluted in three steps, first with 80 μl of digestion buffer, then with 80 μl of 0.2% formic acid, and finally with 80 μl of 50% acetonitrile (ACN) containing 0.2% formic acid. All peptides were dried down in a vacuum concentrator. Peptide determination was performed for all samples using Pierce Quantitative Colorimetric Peptide Assay kit (Thermo Scientific), following the manufacturer's instructions.

### 2.4 Nano LC–MS/MS analysis

The nLC–MS/MS analysis was performed on a Q Exactive HF‐X mass spectrometer coupled to a Dionex UltiMate 3000 RSLCnano UPLC system (Thermo Scientific), with an EASY‐Spray ion source. Peptides from CPs and media were injected in triplicates (1.5 and 1 μg, respectively), utilizing two MS methods detailed later. All samples were loaded onto an Acclaim PepMap 100 C18 (75 μm × 2 cm, 3 μm, 100 Å, nanoViper) trap column and separated on an Acclaim PepMap RSLC C18 column (75 μm × 50 cm, 2 μm, 100 Å) (Thermo Scientific) using a flow rate of 300 nl/min, a column temperature of 60°C. A 145‐min gradient was applied for separation, using solvents A (0.1% formic acid) and B (0.1% formic acid in 80% ACN), increasing solvent B from 2% to 25% in 115 min, then to 32% in the next 10 min, and to 45% in 7 min. Finally, the gradient increased to 90% solvent B in 8 min, continuing for another 5 min.

Regarding the MS approach, peptides from the CP were analysed with one data‐dependent acquisition (DDA) and two data‐independent acquisitions (DIA), whereas peptides from the CM were analysed with two DDA and one DIA runs.

The top 20 DDA method was applied with full MS1 scans at *m*/*z* 375–1500, resolution of 120000 (at 200 *m*/*z*), target AGC value of 3 × 10^6^ and maximum injection time of 100 ms. Fragmentation was done with an NCE of 28, and the isolation window was set to 1.2 *m*/*z*. MS2 scans were acquired with a resolution of 15000 (at 200 *m*/*z*), target AGC value of 1 × 10^5^, maximum injection time of 50 ms, ion selection threshold of 8 × 10^3^ and dynamic exclusion of 40 s.

For the DIA analysis, a complete acquisition cycle consisted of 3 MS1 full scans, each followed by 18 MS2 DIA scans with variable isolation windows. MS1 full scans were acquired at *m*/*z* 375–1455, with a resolution of 120000 (at 200 *m*/*z*), target AGC value of 3 × 10^6^ and maximum injection time of 50 ms. The MS2 scans were acquired with a resolution of 30000 (at 200 *m*/*z*), fragmentation with NCE of 28, target AGC value of 1 × 10^6^, automatic maximum injection time, fixed first mass of 200 *m*/*z* and the variable isolation windows were 13.0, 16.0, 26.0 and 61.0 *m*/*z* (with 27, 13, 8 and 6 loop counts, respectively).

See Table S1 for details on biological and technical repeats. Samples were run in a randomized order, in two distinct batches.

### 2.5 Proteomic data processing

#### 2.5.1 Database search

Database search was done on Proteome Discoverer v2.4 using SEQUEST HT search engine combined with spectral library search, using the UniProtKB human database (accessed on 15 January 2019) and Proteome Tools spectral libraries. Dynamic modifications included oxidation of methionine and N‐terminal acetylation, whereas carbamidomethylation of cysteine was implemented as a static modification. Precursor tolerance was set to 10 ppm and fragment mass tolerance was set to 0.02 Da. Additionally, a maximum of two missed cleavages were allowed, and a false discovery rate (FDR) of 1% was set both on peptide and protein levels. For protein quantitation, the top three average methods were used (i.e. it was calculated based on the average of top three peptide abundances unique to the protein group).

#### 2.5.2 Post‐processing

The data‐processing steps were performed separately for the CP and CM samples. Both the processing and the statistical tests were performed in R v.4.2.0^24^ unless specified otherwise. See https://github.com/bszeitz/SCLC_proteomics for details on all used packages.

The raw protein intensities were log_2_‐transformed, and individual measurements were median normalized by centring their intensities around the global median. Measurements from the same batch were then checked for outliers. After removal of low‐quality measurements in the CM data (see Table S1 for details on removed samples), the median intensity of proteins in repeated measurements from the same MS vial and in the same batch was used for further analysis. Proteins with no quantitative values in any samples were removed, leading to the 9570 and 6425 proteins in the CP and CM, respectively. A strong batch effect that was independent from other factors was observed in the data (Figure S1a); therefore, differences in protein expression caused by batch effect were removed using univariate linear regression. Proteins that had no quantitative values in one of the batches were excluded from the batch effect correction. Consequently, the variance explained by the subtype increased (Figure S1b), and the technical and biological replicates from the same cell line showed high similarity according to unsupervised hierarchical clustering results (Figure S1c). All replicates from the same cell line were, therefore, averaged by taking their arithmetic mean.

For statistical analyses, the expression table was filtered for proteins with min. 80% valid values across samples, resulting in 8405 and 5408 proteins in the CP and CM, respectively. Missing value imputation was performed in Perseus v.1.6^25^ using imputation based on normal distribution (width = 0.3, down shift = 1.8). The protein intensity histogram per sample, also highlighting the frequency distribution of imputed values, is shown on Figure S1d for the samples with the highest missing value content. Additionally, potential ‘on/off proteins’ were also sought out during the filtering step. The ‘on/off proteins’ were defined as proteins that were present in min. 85% of the samples in one subtype and simultaneously, in max. 15% of the samples in the other subtypes (‘on’), or in max. 15% of the samples in one subtype and simultaneously, in min. 85% of the samples in the other subtypes (‘off’). These proteins, together with the filtered and imputed expression table elements, were also examined as part of the analysis of subtype‐specific proteins.

#### 2.5.3 Annotation of secreted, cell‐surface, plasma and ‘druggable’ proteins

To map secreted proteins from the list of identified proteins, three secretome databases were utilized: Human Protein Atlas (The Human Protein Atlas v20.1 and Ensembl v92.38, retrieved on 11 April 2021),^26^ SPRomeDB (accessed on 7 April 2021)^27^ and MetazSecKB (accessed on 7 April 2021).^28^ These databases were created using curated experimental evidence and a number of prediction tools that do not completely overlap. All proteins mentioned in at least two databases were considered secreted proteins. In sum, 422 and 636 secreted proteins were detected in the CP and CM, respectively, out of which 295 and 514 were also quantified in min. 80% of the samples.

Additionally, information from the Human Protein Atlas (The Human Protein Atlas v21.1, https://www.proteinatlas.org/humanproteome/blood+protein, accessed on 23 July 2022) was used to annotate proteins detectable in human blood plasma by immunoassay, MS or proximity extension assays. This was supplemented with a list of proteins actively secreted into the blood (https://www.proteinatlas.org/search/sa_location%3ASecreted+to+blood). In total, 3076 and 3126 plasma proteins were identified in the CP and CM, respectively, of which 2763 and 2823 were quantified in at least 80% of the samples. Among the proteins actively secreted in the blood, 229 and 334 were found in the CP and CM, respectively, and 172 and 278 proteins were quantified in min. 80% of the samples.

Two databases were downloaded to retrieve a list of cell‐surface proteins, namely The Cancer Surfaceome Atlas^29^ (http://fcgportal.org/TCSA/Download.php, accessed on 28 July 2022) and the in silico human surfaceome by Bausch‐Fluck et al.^30^ (http://wlab.ethz.ch/surfaceome/, accessed on 28 July 2022). In the CP and CM, we found a total of 682 and 549 cell‐surface proteins that are included in both databases, of which 477 and 416 were quantified in at least 80% of the samples.

To annotate ‘druggable’ proteins with subtype specific profiles, we used The druggable proteome database available at the Human Protein Atlas website (https://www.proteinatlas.org/humanproteome/tissue/druggable, accessed on 22 October 2021), which contains the protein targets of the current Food and Drug Administration (FDA)‐approved drugs, including enzymes, transporters, ion channels, and receptors. Functional annotations were collected from the UniProt^31^ website (https://www.uniprot.org/, Release 2022_03) for selected proteins, and the list of FDA‐approved drugs that directly interact with those proteins as part of their mechanism of action was gathered from the DrugBank database (www.drugbank.ca).^32^

### 2.6 External data retrieval and processing

#### 2.6.1 Transcriptomic data of SCLC tissue samples

The mRNA data of SCLC tissue samples was accessed from cBioPortal^33^, ^34^ on 24 May 2021, downloading George et al.’s work.^8^ Only samples that were also categorized by Rudin et al.^6^ were further analysed (52 samples out of 81). This resulted in 38 SCLC‐A, 5 SCLC‐N, 7 SCLC‐P and 2 SCLC‐Y samples. These 52 samples’ Fragments per kilobase million (FPKM) values were utilized to perform the single‐sample gene set enrichment analysis (ssGSEA), as well as the log‐transformed mRNA *Z*‐scores (where *Z*‐score was calculated compared to the expression distribution of all samples), were used to assess the expression profile differences between selected transcripts across subtypes (SCLC‐A/N/P/Y).

#### 2.6.2 Genomic and transcriptomic data of SCLC cell lines

Mutation and RNA‐Seq data (gene count data, normalized using RNA‐Seq by Expectation‐Maximization, i.e. RSEM method) of Cancer Cell Line Encyclopedia (CCLE) cancer cell lines were accessed on 28 June 2022 and 23 August 2021, respectively, from the Cancer Dependency Map website, https://depmap.org/portal/.^35^ Compared to our set of cell lines, only five cell lines are not included in the mutation table (GLC4, HLHE, H1882, N417, H372), and two additional cell lines are not present in the RNA sequencing table (H378 and H1688). The RNA‐Seq data contains the measurements from 50 SCLC cell lines, which were also categorized into subtypes by Rudin et al.^6^ (26 SCLC‐A, 12 SCLC‐N, 4 SCLC‐P and 8 SCLC‐Y). The transcriptomic data was further processed via limma R package v.3.46.0,^36^ following the standard RNA‐Seq data‐processing steps. This included normalization factor calculation with default settings, filter of low‐expressed genes (only genes with minimum one expression value equal to or higher than 50 should be kept; fulfilled by 9237 genes) and voom transformation with default settings where model matrix included the subtype assignment. This was followed by differential expression analysis described in detail in Section 2.7. Log_2_ CPM counts were calculated using the ‘cpm’ function from edgeR v.3.32.1^37^, ^38^, ^39^ in which prior count was set to 3 (i.e. the average count to be added to each observation to avoid zeros in the dataset during log_2_‐transformation). This expression matrix was used to display the differential expression of selected transcripts across subtypes.

#### 2.6.3 Drug sensitivity data of SCLC cell lines

CancerRxGene drug sensitivity data^40^ (Release 8.3, available from June 2020) was downloaded from the FTP Server of the Wellcome Sanger Institute (ftp://ftp.sanger.ac.uk/pub/project/cancerrxgene/releases/current_release/) on 18 July 2022. Both the Genomics of Drug Sensitivity in Cancer 1 (GDSC1) and GDSC2 datasets were investigated. In total, the subtype classification of 38 SCLC cell lines is known,^6^ of which 20 were also measured by proteomics in our study (the missing cell lines: DMS153, GLC4, H1882, H372, HLHE, N417). The logarithmic IC50 values of these cell lines for selected drugs were then compared across the subtypes or were correlated with protein abundance.

### 2.7 Statistics and bioinformatics

#### 2.7.1 Differential expression analyses

Differential expression analyses for the proteomic data were performed via ANOVA, followed by multiple testing correction (Benjamini–Hochberg, BH) of ANOVA *p*‐values and Tukey's honestly significant difference (HSD) post hoc tests. Comparisons were done between culture types (adherent, suspension) and subtypes (SCLC‐A, ‐N, ‐P, ‐Y). A protein was considered significant for a comparison if both the ANOVA FDR and the corresponding pairwise Tukey's HSD test *p*‐value were less than 0.05. Subtype‐specific proteins were selected based on the following criteria: ANOVA FDR < 0.05, as well as Tukey's HSD test *p* < 0.05 for all comparisons of the subtype of interest against the other subtypes. Differential expression analysis for the CCLE RNA‐Seq data was performed via limma by fitting a linear model for the six subtype comparisons, followed by Empirical Bayes smoothing of standard errors. Multiple testing correction with the BH method was applied and FDR < 0.05 was considered significant. For the transcriptomic dataset by George et al., pairwise Wilcoxon tests were performed on the *Z*‐scored values to check gene expression differences between the subtypes. Significance level was set to nominal *p* < 0.05.

Overrepresentation analyses (ORAs) were conducted using clusterProfiler v.3.18.1^41^, ^42^ and ReactomePA v.1.34.0.^43^ The default list of human genes was used as background.

#### 2.7.2 Neuroendocrine and epithelial–mesenchymal transition scores

Two previously published gene sets were used to introduce a scoring system for the NE and epithelial–mesenchymal transition (EMT) characteristics of cell lines. For NE characteristics, a 50‐gene NE signature built for SCLC cell lines described by Zhang et al.^7^ was examined. The number of NE (i.e. markers involved in NE differentiation) and non‐NE markers present in our CP data was 19 and 17, respectively (Figure S3a). NE score was calculated the following way for each cell line:

NE score = (Mean *Z*‐score of NE markers) − (Mean *Z*‐score of non‐NE markers).

Similarly, a gene signature with 22 epithelial and 15 mesenchymal genes published by Kohn et al.^44^ was examined, from which 12 and 10 corresponding proteins were quantified in our CP data (Figure S3b). EMT score was calculated the following way for each cell line:

EMT score = (Mean *Z*‐score of mesenchymal markers) − (Mean *Z*‐score of epithelial markers).

#### 2.7.3 Consensus clustering

Samples were grouped in an unsupervised manner using the consensus clustering algorithm, implemented in the ConsensusClusterPlus R package v.1.54.0.^45^ The basis of clustering was the samples’ global protein expression profile (filtered and imputed) in the CP dataset, which was further restricted to only those proteins that have an SD above 1.25. This list of proteins included protein products of *POU2F3* and *YAP1*, both showing large SD (SD = 1.40 and 2.05, respectively), whereas *NEUROD1* did not show sufficiently large variance (SD = 0.50), and *ASCL1* was not quantified in enough samples to be included in the list. The resulting proteome data (including the expression of 1157 proteins without *Z*‐score normalization) were resampled 1000 times via the bootstrap method with a probability of 0.8 for selecting any item (i.e. sample) and any feature (i.e. protein). The bootstrap sample datasets were clustered using the partitioning around medoids method with the Pearson distance and complete linkage, exploring the range of two to seven clusters. The visual inspection of consensus matrices, as well as the silhouette plots for *K* = 4 and *K* = 5 revealed that a cleaner separation of the clusters can be achieved with *K* = 4; moreover, the average silhouette width was higher for *K* = 4. Based on these observations, and considering the relatively low number of samples, consensus clustering with *K* = 4 was used as sample grouping. Graphical outputs from the ConsensusClusterPlus R package and silhouette plots for *K* = 4 and *K* = 5 are shown in Figure S4a–c. Silhouette information was computed using the ‘silhouette’ function from R package ‘cluster’ v.2.1.1.

#### 2.7.4 Gene set enrichment analysis

Pre‐ranked GSEA (pGSEA) was performed via the ‘GSEA’ function of the clusterProfiler R package for all six subtype comparisons. As input, the hallmark,^46^ KEGG,^47^ Reactome,^48^ Gene Ontology biological process^49^, ^50^ and oncogenic curated gene sets were used, downloaded from the Molecular Signatures Database (MSigDB) v.7.4,^51^, ^52^ as well as a ranked list of genes/proteins (based on the multiplication of the FC and −log_10_ (*p*‐value of pairwise test), calculated for all proteins/genes used in the statistical analyses). The pairwise test was the Tukey's HSD post hoc test for proteomics, whereas for CCLE RNA‐Seq, the *p*‐values were derived from the moderated *t*‐test statistic. The BH method was used for *p*‐value adjustment in pGSEA. To obtain the list of subtype‐characteristic gene sets, the following filter was applied on the pGSEA results: *p* < 0.01 in all comparisons of the subtype of interest against the other subtypes with an unequivocal sign (either positive or negative) normalized enrichment score (NES). Gene sets with a *p* < 0.01 for at least one comparison are listed in Table S4. The relationship between proteomic and transcriptomic results was examined on these subtype‐specific gene sets as follows: if a gene set in one dataset (either proteomic or transcriptomic) was found to be characteristic for a subtype, and the *p*‐value of the same gene set was less than 0.1 in relevant comparisons of the other dataset, as well as its NES values showed the same direction, the gene set was declared significant in both proteomics and transcriptomics. In contrast, if the gene set in the other dataset did not meet the previous criteria, the gene set was declared significant in only one dataset. A visual explanation for the filtering steps can be seen in Figure S5 and a summary of the obtained results in Table S4.

ssGSEA^53^ was performed on the transcriptomic dataset by George et al., using only transcripts with sum FPKM higher than 50, and only one transcript for each gene was retained (the transcript with the highest sum FPKM value). This resulted in an expression matrix with 13542 transcripts. It should be noted that different transcripts from the same gene may have different biological functions; however, we cannot preserve such depth of information in this analysis. The NES was calculated for subtype‐specific gene sets as defined earlier (the list can be accessed in Table S4), with the script available on https://github.com/broadinstitute/ssGSEA2.0 (accessed on 05 February 2022). The parameters were set as follows: sample.norm.type = ‘rank’, weight = 0.75, statistic = ‘area.under.RES’, output.score.type = ‘NES’, nperm = 1000, min.overlap = 5, and correl.type = ‘z.score’. The gene sets were then filtered for the most representative gene sets (Table S4), and ssGSEA results for these are depicted in Figure S6. For visualization purposes, NES values of each gene set were min.–max. scaled across the samples.

#### 2.7.5 Sparse partial least squares discriminant analysis

The sparse partial least squares discriminant analysis (sPLS‐DA) was performed via the mixOmics R package v.6.14.0,^54^ following the guidelines provided on its website (http://mixomics.org/case‐studies/splsda‐srbct/, accessed on 27 July 2021). The full CP and CM data (filtered and imputed) were analysed separately. First, a PLS‐DA model was fitted with 10 components to evaluate the performance and to select the optimal number of components for the final model. The performance plot showed that five and four components with centroid distance measure are sufficient for good performance (0.169 and 0.476 balanced error rate) in the CP and CM data, respectively (Figure S7a). Then sPLS‐DA model was tuned, estimating the classification performance with respect to the number of selected variables in the model. Maximum number of components was set to five and four in CP and CM, respectively, threefold cross‐validation was used which was repeated 50 times, and centroid distance measure was selected. As a result, the settings of the final model were the following in CP: (1) optimal number of components is 3, (2) the number of proteins to select on each component are 45, 30 and 7. In the case of CM, the settings of the final model: (1) optimal number of components is 2; (2) the number of proteins to select on each component are 8 and 15. The sample plots of the first three components in CP (Figure S7b) showed that SCLC‐Y is well separated on the first component, SCLC‐A and ‐N are distinguishable on the second component and adding the third component further discriminates SCLC‐P from the rest. Similarly, the first two components were visualized for CM, displaying that SCLC‐Y is best separated on the first component, whereas SCLC‐A/P can be distinguished from SCLC‐N on the second component. The classification performance of these final sPLS‐DA models were 0.113 and 0.484 with centroid distance for CP and CM, respectively (Figure S7c). Additionally, selected proteins in each component with their loading weight and stability (i.e. ratio of how many times it was selected across the cross‐validation runs) were extracted. The selected proteins are shown in Supplementary Figure S8. To select the transcripts showing the best match with proteomic results, transcripts were ranked based on the multiplication of the fold change (FC) and −log_10_ (*p*‐value of pairwise test).

#### 2.7.6 Visualizations

Individual clustering settings for heat map visualizations were hierarchical clustering, Euclidean distance and complete linkage, unless specified otherwise in the figure legends. Heat maps were plotted using ComplexHeatmap R package v.2.6.2.^55^ Principal component analyses (PCAs) and principal variance component analyses (PVCAs) were done on the *Z*‐scored protein expression tables. PCA biplots were drawn using the ggbiplot R package v.0.55 (https://github.com/vqv/ggbiplot). Further visualizations were done using R v.4.2.0 and GraphPad Prism v.8 for Windows, GraphPad Software, San Diego, California USA, www.graphpad.com.

## 3 RESULTS

### 3.1 Molecular heterogeneity of SCLC cell lines detected via proteomics

We characterized 26 cell lines derived from primary or metastatic human SCLC lesions (Table 1) using label‐free proteomic analysis. A total of 10161 proteins were identified and quantified (9570 and 6425 proteins in CP and CM, respectively), and the majority of these proteins were quantified in minimum 80% of the samples (8405 and 5408 proteins in CP and CM, respectively). Altogether, we annotated 699 secreted proteins, 800 cell‐surface proteins, 3440 proteins detectable in human blood plasma, of which 367 are actively secreted into the blood, and 289 ‘druggable’ proteins in our cell lines. The CM consisted of relatively more secreted and plasma proteins, highlighting its added value in the search for potential blood‐based biomarkers.

**FIGURE 1:**
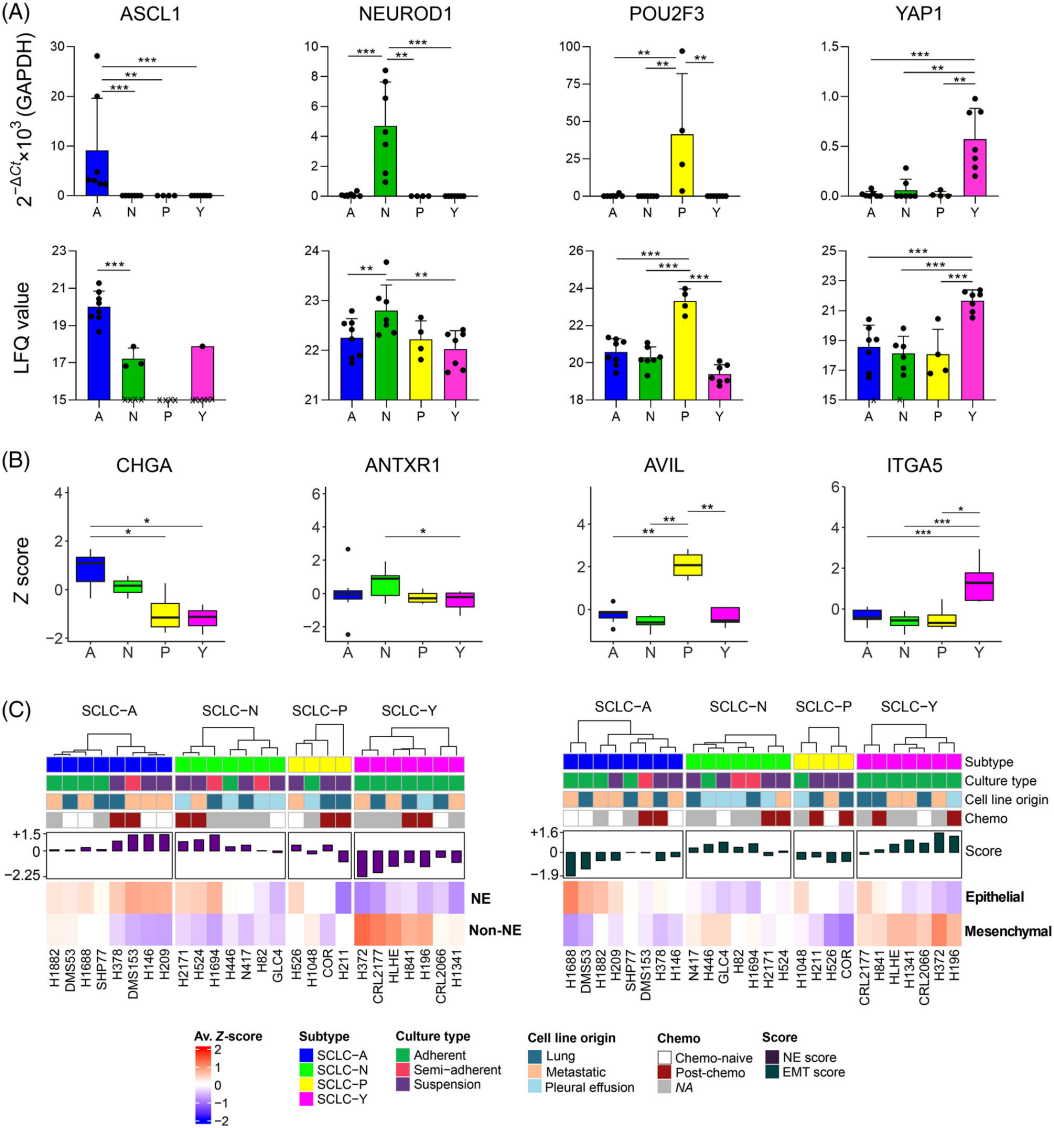
Proteomic analysis of small‐cell lung cancer (SCLC) cell lines highlights molecular heterogeneity: (A) The mRNA expression of key genes *ASCL1*, *NEUROD1*, *POU2F3* and *YAP1* to determine the molecular subtypes (top). Data is shown as mean ± SD of the 2^−Δ^ *^Ct^* × 1000 value, normalized to glyceraldehyde 3‐phosphate dehydrogenase (*GAPDH*). Each dot represents one cell line and is the mean of two biological replicates performed in triplicates. The significance of Mann–Whitney *U* tests is indicated above the boxplots (*p < 0.05; **p < 0.01; ***p < 0.001). Bottom panel shows label‐free quantitation (LFQ) values derived from the proteomic analysis, shown as mean ± SD of each cell line, for each defined subtype. Missing values are indicated by an *x*. The significance of independent *t*‐tests is indicated above the boxplots (*p < 0.05; **p < 0.01; ***p < 0.001); (B) protein expression profile of four well‐known subtype markers (from left to right: *CHGA*, *ANTXR1*, *AVIL* and *ITGA5*, markers for SCLC‐A, ‐N, ‐P and ‐Y, respectively). The significance of independent *t*‐tests is indicated above the boxplots (*p < 0.05; **p < 0.01; ***p < 0.001); (C) mean *Z*‐score values for neuroendocrine (NE) and non‐NE markers in each cell line (left), and mean *Z*‐score values for epithelial and mesenchymal markers (right)

First, according to their *ASCL1*, *NEUROD1*, *POU2F3* and *YAP1* mRNA expression patterns – we grouped the cell lines into one of the four respective subgroups: SCLC‐A, SCLC‐N, SCLC‐P and SCLC‐Y (eight, seven, four and seven cell lines, respectively) (Figure 1A, upper panel). These transcription factors also showed increased protein levels in their respective subtype (Figure 1A, lower panel).

In general, we identified several members of the Myc proto‐oncogene (MYC) family universally expressed across our samples, such as MYC and protein L‐Myc (MYCL),^6^ or the transcription activator nuclear factor 1 B‐type (Figure S2a).^56^ The protein products of *TP53* and *RB1* genes, which are well known to be genetically altered in SCLC,^57^, ^58^, ^59^ were quantified in 100.0% and 88.5% of the cell lines, respectively, independent of the previously described *TP53* and *RB1* mutational status of the cell lines^35^ (Figure S2b). Several well‐accepted subtype markers^3^ showed the expected protein expression profile across the subtypes (Figure S2c), such as chromogranin‐A (SCLC‐A marker), anthrax toxin receptor 1 (SCLC‐N marker), advillin (SCLC‐P marker) and multiple integrins (SCLC‐Y markers) (Figure 1B).

The cell lines were further characterized by their NE and EMT features. NE scores built from 19 NE and 17 non‐NE markers,^7^ and EMT scores based on 12 epithelial and 10 mesenchymal markers,^44^ were calculated for each cell line (Figure S3a,b). The mean protein abundances of NE, non‐NE, epithelial and mesenchymal markers in each sample are shown in Figure 1D, whereas the mean NE and EMT scores across the subtypes are depicted in Figure S3c. As expected, most SCLC‐A cell lines expressed NE and epithelial markers more strongly than non‐NE and mesenchymal markers (*M*_NE score_ = 0.71, *M*_EMT score_ = −0.70). SCLC‐N was found to be a rather NE subtype with mixed epithelial–mesenchymal characteristics (*M*_NE score_ = 0.56 and *M*_EMT score_ = 0.39). SCLC‐P exhibited moderate non‐NE characteristics in our dataset (i.e. lower than SCLC‐A and ‐N, but higher than SCLC‐Y); however, a high expression of epithelial markers was detected (M_NE score_ = −0.05 and M_EMT score_ = −0.61) in this subtype. In contrast, SCLC‐Y cell lines exhibited prominent non‐NE and mesenchymal traits (M_NE score_ = −1.35 and M_EMT score_ = 0.75). In line with these findings, delta‐like protein 3 (DLL3) protein, an inhibitory Notch pathway ligand,^60^ was expressed in decreasing amounts from SCLC‐A to SCLC‐Y, indicating the Notch pathway's gradual activation (Figure S2c).

Comparing NE and EMT scores between cell lines with different properties, such as culture type, cell line origin and treatment with chemotherapy, we found that adherent cell lines harbour significantly lower NE scores than the non‐adherent ones (Figure S3d–f). In addition, a significant negative correlation was observed between NE and EMT scores (Figure S3g).

### 3.2 Manifestation of heterogeneous in vitro growth characteristics in the proteome

**FIGURE 2:**
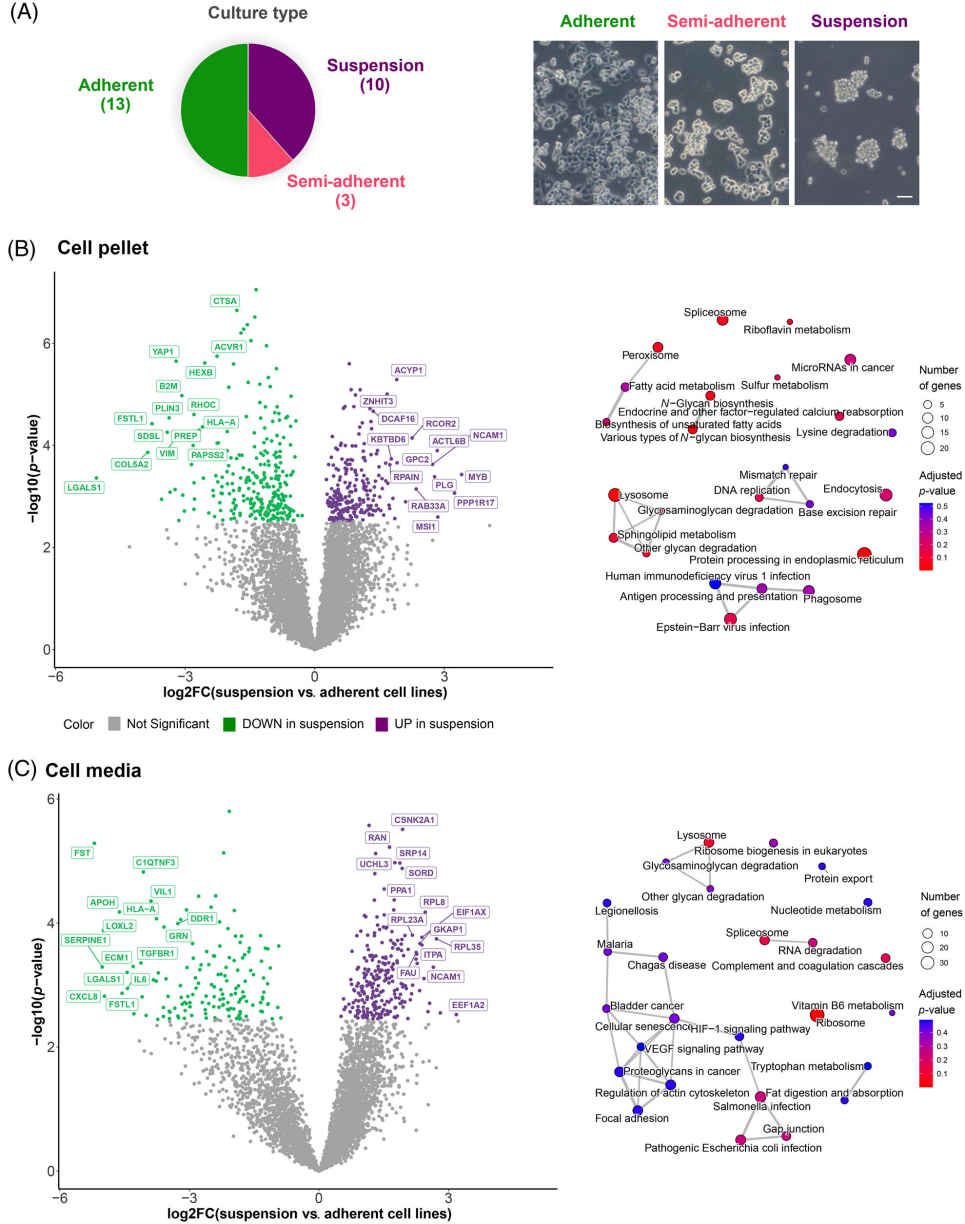
In vitro growth characteristics mirrored in the proteome: (A) Pie chart and representative images (taken on a Zeiss Axiovert 40 C microscope) of the different culture types of small‐cell lung cancer (SCLC) cell lines (*n* = 26). Scale bar = 100 μm; (B) volcano plot depicting the results of differential expression analysis results between suspension and adherent cell lines (left) with the corresponding enrichment map of the overrepresented KEGG pathways (right) in the cell pellet (CP) data; (C) volcano plot depicting the results of differential expression analysis between suspension and adherent cell lines (left) with corresponding enrichment map of the overrepresented KEGG pathways (right) in the culture media (CM) data

Although maintained in the same in vitro conditions, the cell lines showed significantly different growth characteristics. Specifically, out of the 26 cell lines, 10 (38.5%) grew in suspension, 3 (11.5%) in a semi‐adherent form and the other 13 (50.0%) grew adherent on the plastic (Figure 2A). Adherent and non‐adherent cell lines showed clearly distinct protein expression profiles (Figure 2B,C). In total, 270 and 148 proteins were significantly (BH adjusted *p* < 0.05) downregulated in suspension cell lines compared to adherent cell lines in the CP and CM, respectively, whereas 244 and 244 proteins were significantly upregulated in suspension cell lines in the CP and CM, respectively (Figure 2B,C, left). An ORA of differentially expressed proteins, separately for CP and CM but combining up‐ and downregulated proteins, showed that KEGG pathways such as protein processing in endoplasmic reticulum, lysosome and glycosaminoglycan degradation were significantly (*p* < 0.05) enriched both in CP and CM, as well as other pathways such as endocytosis in CP and gap junction in CM were overrepresented, thereby supporting the phenotypic cell line differences on protein level (Figure 2B,C, right and Table S2).

### 3.3 Proteome‐based SCLC subgroups match with mRNA‐based subtyping

**FIGURE 3:**
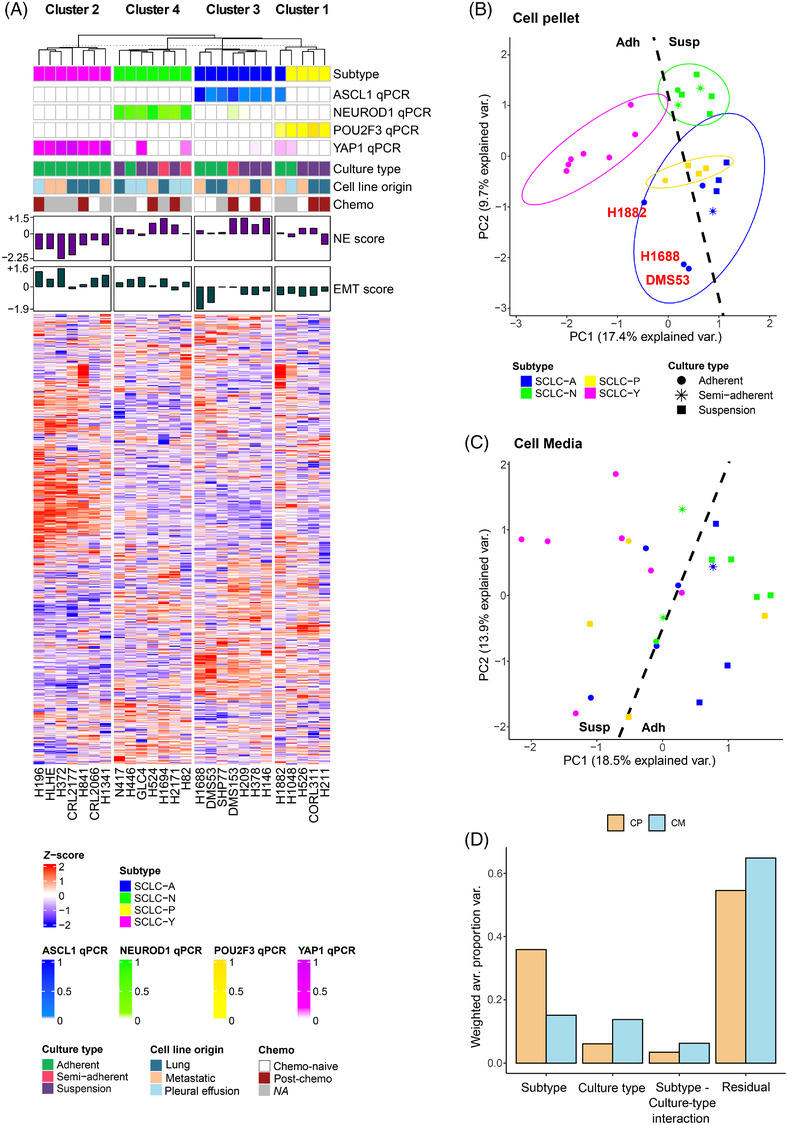
The mRNA‐based classification of small‐cell lung cancer (SCLC) subtypes correlates with proteomic data: (A) Heat map of consensus clustering results using proteins from the cell pellet (CP) data showing high variation (>1.25 SD). Samples are sorted according to their cluster assignments and their representative protein expression profiles are shown; (B) principal component analyses (PCA) plot of the CP data using the most variable (>1.25 SD) proteins. Cell lines are coloured according to the subtype and shape corresponds to culture type. Normal data ellipses are also drawn for each subtype (probability = 68%); (C) PCA plot of the culture media (CM) data using only the most variable (>1.25 SD) proteins. Cell lines are coloured according to the subtype and shape corresponds to culture type; (D) principal variance component analysis (PVCA) reveals the contribution of subtype and culture type to the proteomic profile differences in CP and CM. The residual variation (noted as ‘residual’) represents the remaining biological and technical variance in the dataset which could not be attributed to the abovementioned factors.

To investigate whether proteomic subtyping correlates with the mRNA‐based classification, unsupervised consensus clustering of the CP samples was performed based on the most variable proteins (Figure S4a–c). The analysis revealed four clusters in the proteomic data, which agrees with the mRNA‐based subtyping (Figure 3A). A discrepancy was detected in only one cell line (H1882), which was classified in the SCLC‐A subgroup according to the qPCR data and in the SCLC‐P subset based on the proteomic results. Of note, this cell line also displayed a higher *POU2F3* mRNA expression than other SCLC‐A cell lines (Figure 3A). Additionally, two adherent SCLC‐A samples (H1688, DMS53) were rather separated from their group members (Figure S4c). Notably, SCLC‐Y samples exhibited the most distinct protein expression profile. The previous observations are also well reflected on a PCA plot of the CP samples (Figure 3B). The CM samples, on the other hand, exhibit a rather heterogeneous expression profile based on their most variable proteins, and the PCA plot showed no apparent separation according to the mRNA‐based subtype classification (Figure 3C). Correspondingly, PVCA revealed that the molecular subtypes were less prominent contributors to protein expression variability in CM compared to CP (Figure 3D). Furthermore, culture type was identified as an important contributor to the proteomic profiles of SCLC cell lines, which was more pronounced in CM.

### 3.4 Multi‐omic portraits of SCLC subtypes outline potential subtype‐specific vulnerabilities

According to the high concordance between mRNA‐ and proteome‐based subtypes, we used the mRNA‐based classification system in differential expression analyses between subtypes. We found 367 and 34 subtype‐specific proteins (ANOVA BH adjusted *p* < 0.05, Tukey's HSD post hoc *p* < 0.05), the levels of which differ in a given subtype compared to all the three other subtypes in the CP and CM data, respectively (Table S3). This also included four proteins with on/off characteristics in the CP data, namely achaete‐scute homolog 1 (ASCL1; ‘on’ in SCLC‐A), regulator of G‐protein signalling 22 (RGS22; ‘on’ in SCLC‐P), neurexophilin‐4 and puratrophin‐1 (NXPH4 and PKHG4; ‘off’ in SCLC‐Y). All subtype‐specific proteins (including those from CP and CM) were then subjected to the pathway analysis (Table S3).

In order to more systematically investigate the underlying biology of SCLC subtypes, in addition to identifying subtype‐specific proteins, we also performed a pathway‐based comparison of subgroups using pGSEAs. We looked for significant pathways that were concordantly activated or suppressed in a certain subtype compared to the other three subtypes. To do this, all pairwise subtype comparisons were performed, using the full list of quantified proteins (*n* = 8405) from the CP data. Furthermore, we performed the same analysis on the RNA‐Seq data of 50 SCLC cell lines from CCLE^35^ (*n* = 9237 genes) and finally assessed the relationship between proteomic and transcriptomic results (Table S4). These steps are described in detail in Section 2 and Figure S5.

**FIGURE 4:**
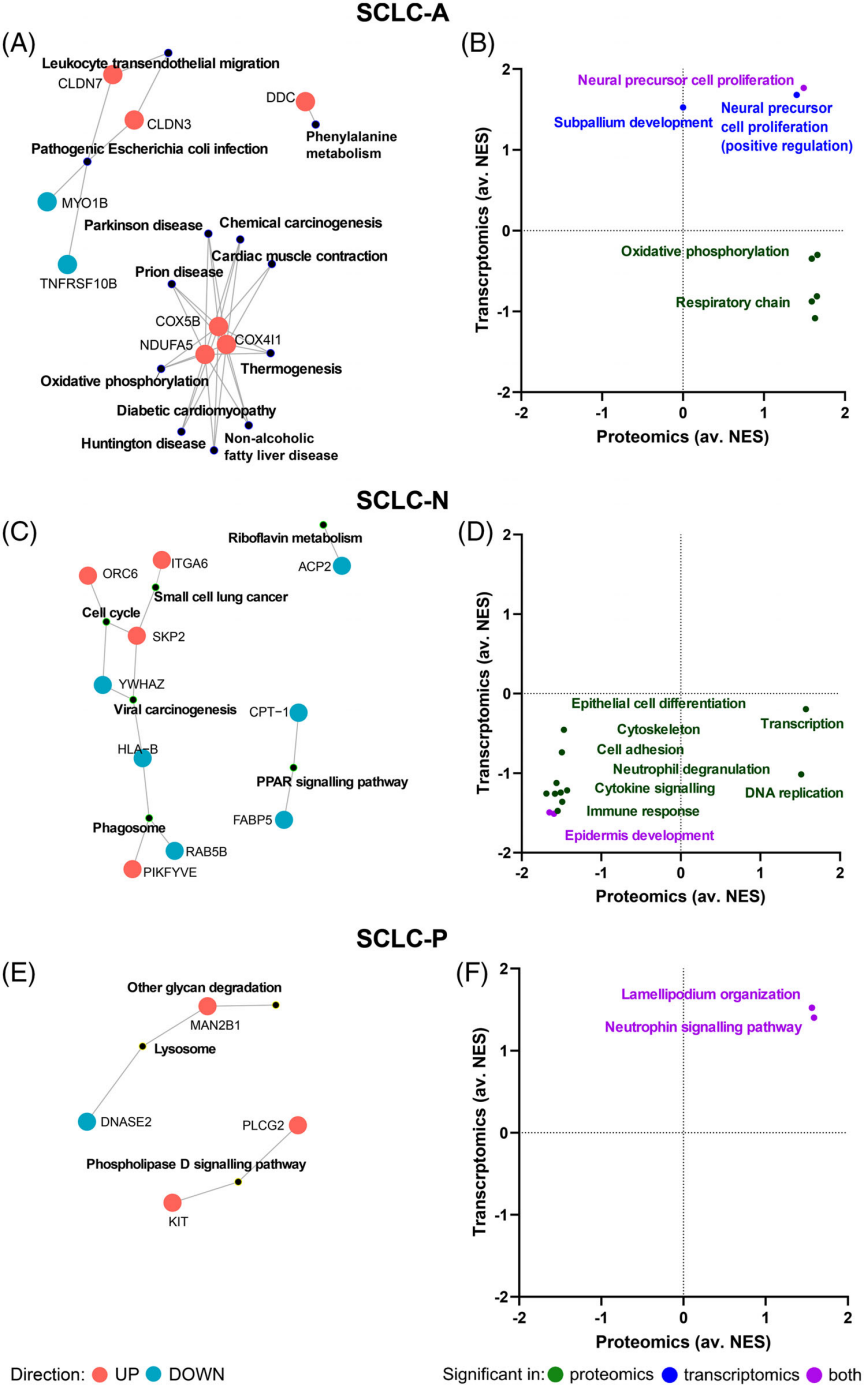
Subtype‐specific biological processes in small‐cell lung cancer (SCLC)‐A/N/P. Significantly overrepresented KEGG pathways (*p* < 0.05) derived from the list of subtype‐specific proteins, as well as the members of these pathways (red and blue colours mean up‐ and downregulated in the given subtype compared to the other subtypes, respectively) are shown on the left side of the panel. The characteristic gene sets for each subtype, as determined by pre‐ranked gene set enrichment analysis (pGSEA), are shown on the right side of the panel. The *x* axis indicates the average normalized enrichment score (av. NES) in proteomics for comparisons SCLC‐A versus ‐N/P/Y, SCLC‐N versus ‐A/P/Y, or SCLC‐P versus ‐A/N/Y, whereas *y* axis indicates the av. NES in transcriptomics. Dots refer to individual gene sets, which are summarized by keywords. Gene set activation or suppression supported by both omic data is shown in purple, whereas green and blue means gene sets supported only by proteomics or transcriptomics, respectively. (A and B) SCLC‐A, (C and D) SCLC‐N and (E and F) SCLC‐P

Significantly overrepresented (*p* < 0.05) KEGG processes in SCLC‐A according to subtype‐specific proteins (*n* = 33) include oxidative phosphorylation (OXPHOS), as well as phenylalanine metabolism and leukocyte transendothelial migration (Figure 4A). Consistent with this, pGSEA results also showed upregulation of OXPHOS and respiratory chain elements based on proteomic data. Positive regulation of neural precursor cell proliferation was supported by both datasets, whereas transcriptomics showed the activation of the subpallium development gene set in SCLC‐A (Figure 4B).

Subtype‐specific proteins in SCLC‐N (*n* = 54) contributed to the significant overrepresentation of KEGG pathways, such as cell cycle, phagosome, riboflavin metabolism and peroxisome proliferator‐activated receptor signalling pathway (Figure 4C). SCLC‐N could be further characterized by the suppression of epidermis development processes based on pGSEA. Proteomic data also outlined the downregulation of immune response, cytokine signalling, cell adhesion and cytoskeleton organization, as well as upregulation of transcription and DNA replication (Figure 4D).

Considering SCLC‐P specific proteins (*n* = 32), three significantly enriched KEGG pathways were detected, namely phospholipase D signalling, lysosome as well as other glycan degradation (Figure 4E). Furthermore, pGSEA showed activation of the neurotrophin signalling pathway and the lamellipodium organization gene set in SCLC‐P (Figure 4F).

**FIGURE 5:**
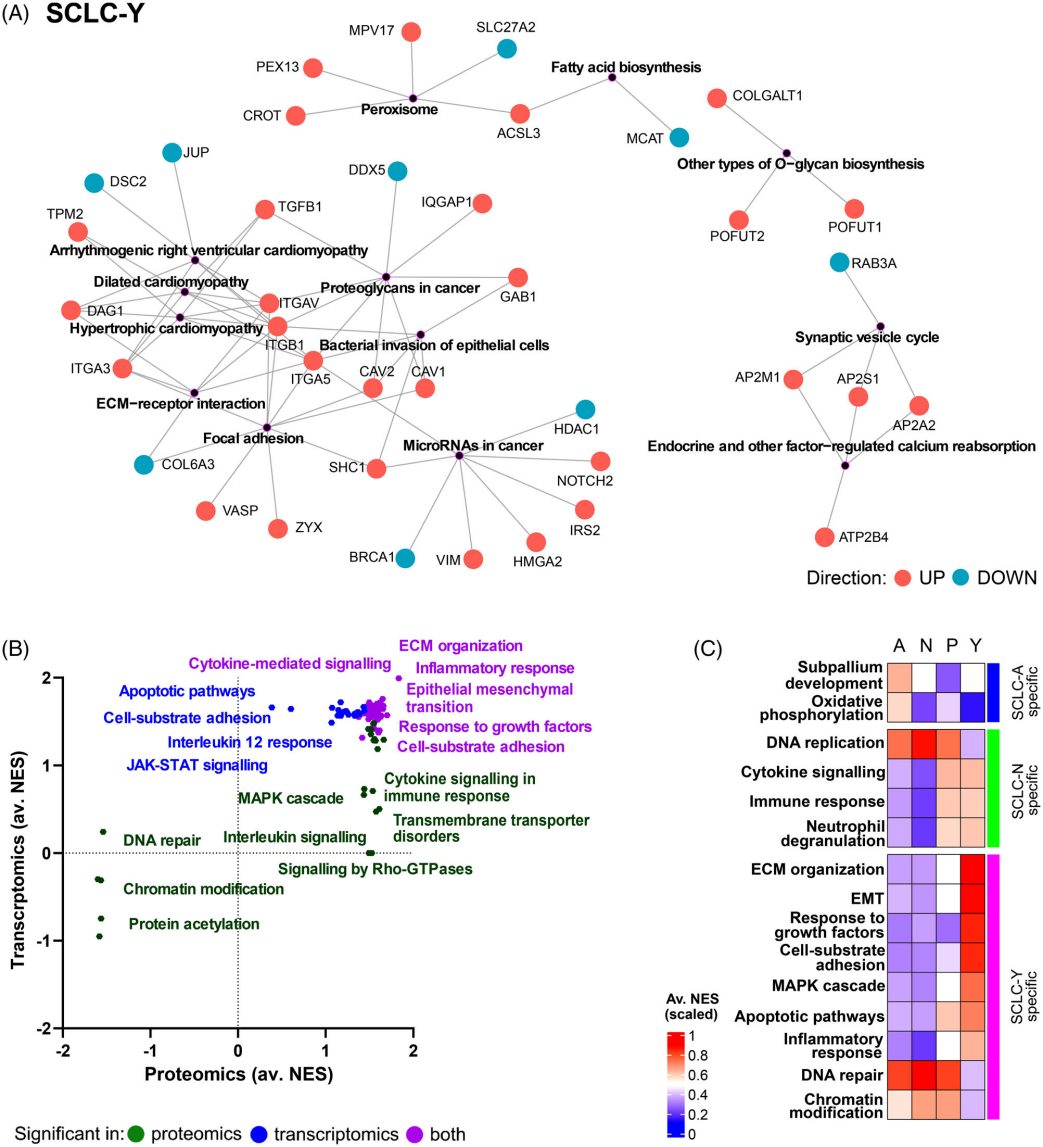
Subtype‐specific biological processes in small‐cell lung cancer (SCLC)‐Y and processes verified using tissue transcriptomics: (A) Significantly overrepresented KEGG pathways (*p* < 0.05) derived from the list SCLC‐Y specific proteins, as well as the members of these pathways (red and blue means up‐ and downregulated in SCLC‐Y, respectively); (B) the characteristic gene sets for SCLC‐Y determined by pre‐ranked gene set enrichment analysis (pGSEA). The *x* axis indicates the average normalized enrichment score (NES) in proteomics for comparisons SCLC‐Y versus ‐A/N/P, whereas the *y* axis indicates the average NES in transcriptomics. Dots show the individual gene sets, which are then summarized by keywords. Colouring is based on whether the gene set activation or suppression was detected by both proteomics and transcriptomics (purple), or only by proteomics (green) or transcriptomics (blue). (C) The average value of NESs per subtype for SCLC tissue samples, derived from single‐sample gene set enrichment analysis (ssGSEA) for some representative gene sets where the subtype specificity was also supported by the tissue data.

Regarding SCLC‐Y, multiple KEGG pathways were significantly overrepresented in the context of subtype‐specific proteins (*n* = 271), such as extracellular matrix (ECM)‐receptor interaction, focal adhesion, spliceosome, peroxisome or *O*‐glycan biosynthesis (Figure 5A). Similarly, a bulky list of processes showed upregulation in SCLC‐Y compared to other subtypes according to pGSEA, such as ECM organization, cytokine‐mediated signalling, interleukin signalling, inflammatory response, EMT, response to growth factors, cell‐substrate adhesion and mitogen‐activated protein kinase (MAPK) cascade. Transcriptomic data showed an activation of apoptotic pathways and the Janus kinase‐signal transducer and activator of transcription signalling, whereas proteomics revealed the upregulation of signalling by Rho‐GTPases, as well as activation of transmembrane transporter disorder‐related processes. Moreover, DNA repair, protein acetylation and chromatin modification were found downregulated in this subtype, as detected by the proteomic data (Figure 5B).

In order to verify the subtype‐specificity observed in our cell lines in SCLC tissues as well, we investigated the behaviour of representative gene sets (*n* = 33) of subtype‐specific processes outlined earlier in the SCLC tissue transcriptomic dataset published by George et al.^8^ ssGSEA identified 22 gene sets for which subtype‐specificity was confirmed to some extent in the tissue data (Figures 5C and S6). Of these, OXPHOS activation in SCLC‐A, upregulated DNA replication and downregulated immune response in SCLC‐N, as well as more active EMT and suppressed DNA repair in SCLC‐Y could be highlighted. Neither of the subtype‐specific processes in SCLC‐P could be verified by tissue transcriptomics.

### 3.5 Proteomic analysis identifies potential diagnostic markers and druggable targets for SCLC subtypes

**FIGURE 6:**
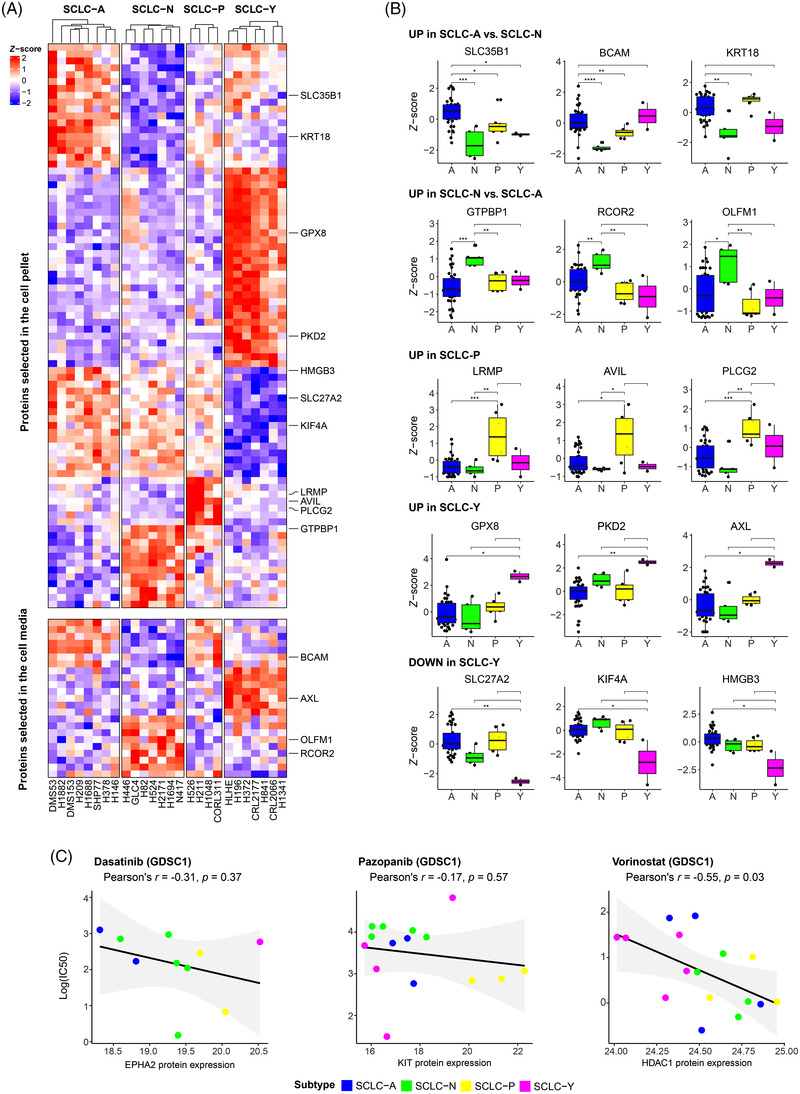
Proteins with diagnostic and therapeutic relevance in small‐cell lung cancer (SCLC) subtypes: (A) The proteins selected by partial least squares discriminant analysis (sPLS‐DA), which are suitable for separating the subtypes based on their expression profile. Results from cell pellet (CP) and culture media (CM) are displayed on the top and bottom heat map, respectively. Proteins with the best matching expression profiles between cell line proteomics and tissue transcriptomics are highlighted; (B) gene expression differences of the best matching transcripts showing subtype‐specific expression profile. The significance of Wilcoxon tests is indicated above the boxplots (*p < 0.05; **p < 0.01; ***p < 0.001); (C) the ln(IC50) values of the cell lines for drugs selected from the Genomics of Drug Sensitivity in Cancer 1 (GDSC1) database (from left to right: dasatinib, pazopanib, and vorinostat), as a function of the measured protein expression. The results of the Pearson correlation analysis are indicated above the scatter plots. Dots are coloured according to the cell line's subtype assignment.

In addition to differential expression analysis, sPLS‐DA (Figure S7) was performed separately for CP and CM to identify the proteins that are most suitable for subtype classifications based on their expression patterns (i.e. potential IHC‐ or blood‐based markers). The analysis resulted in 104 proteins (82 and 23 in the CP and CM datasets, respectively; one protein was detected in both datasets) showing clearly distinct profiles between at least two subtypes (Figure S8). The proteins selected by sPLS‐DA could be separated into the following expression pattern categories: upregulated in SCLC‐A versus ‐N, upregulated in SCLC‐N versus SCLC‐A, upregulated in SCLC‐P (vs. other subtypes), upregulated in SCLC‐Y and downregulated in SCLC‐Y (Figure 6A). Of note, 35 and 17 out of the 104 proteins showed either clear up‐ or downregulation in the SCLC‐Y subtype, respectively. For several markers, the expression pattern was matching with transcriptomic data from SCLC tissues (top three is shown in Figure 6B). Notably, eight of such proteins were found to be overexpressed in SCLC‐Y, including probable glutathione peroxidase 8 (*GPX8*), pyruvate dehydrogenase kinase isoform 2 (*PDK2*) and tyrosine–protein kinase receptor UFO (*AXL)* (Figures 6B and S9a).

Among the proteins confirmed on tissue transcriptome level, six are also detectable in human blood plasma by MS, namely tyrosine–protein kinase receptor UFO (*AXL*, upregulated in SCLC‐Y), basal cell adhesion molecule (*BCAM*, upregulated in SCLC‐A vs. ‐N), GTP‐binding protein 1 (*GTPBP1*, upregulated in SCLC‐N vs. ‐A), keratin‐18 (*KRT18*, upregulated in SCLC‐A vs. ‐N), noelin (*OLFM1*, upregulated in SCLC‐N vs. ‐A) and phospholipase C‐gamma‐2 (*PLCG2*, upregulated in SCLC‐P). The most promising blood‐based biomarker from this list is the protein UFO, which has been previously detected via immunoassay as well.

Finally, we investigated whether among the proteins detected by differential expression analysis or sPLS‐DA (a total of 418 unique proteins), we could find ‘druggable’ proteins^61^ (i.e. drug targets approved by FDA). We identified six of such proteins, listed in Table 2, as potential targets for subtype‐specific therapies: aromatic‐L‐amino‐acid decarboxylase (*DDC*, overexpressed in SCLC‐A), ephrin type‐A receptor 2 (*EPHA2*), integrin alpha‐V and beta‐1 (*ITGAV*, *ITGB1*, overexpressed in SCLC‐Y), histone deacetylase 1 (overexpressed in SCLC‐A/N/P vs. SCLC‐Y), and mast/stem cell growth factor receptor Kit (*KIT*, overexpressed in SCLC‐P). Multiple drugs that directly interact with the aforementioned proteins as part of their mechanism of action could be delineated (Table 2). Moreover, SCLC cell lines have already been tested against seven of these drugs (CancerRxGene database^40^), namely dasatinib (targeting EPHA2), vorinostat (targeting HDAC1), imatinib, pazopanib, sorafenib, sunitinib and tivozanib (all targeting KIT). We therefore investigated whether the subtypes showed differences in sensitivity to these drugs, either in GDSC1 or in GDSC2 datasets (Figure S9b). Generally, we found that lower EPHA2, KIT and HDAC1 protein abundance in our cell lines were indicative of increased resistance to the drugs targeting these proteins (Figure 6C) according to the GDSC1 dataset. This trend was not verified by the GDSC2 dataset for dasatinib; however, median IC50 values of some KIT‐targeting drugs (pazopanib, sunitinib, tivozanib) were lowest in SCLC‐P cell lines, as well as SCLC‐Y subtype's higher resistance to vorinostat could be validated (Figure S9b).

**TABLE 2:** Potentially targetable subtype‐specific proteins

Protein name (gene name)	Dataset	Specificity	Annotation	FDA‐approved drugs with pharmacological action
Aromatic‐l‐amino‐acid decarboxylase (*DDC*)	CP	↑ in A	Enzyme that catalyses dopamine and serotonin synthesis	Benserazide, carbidopa, methyldopa
Ephrin type‐A receptor 2 (*EPHA2*)	CP	↑ in Y	Receptor tyrosine kinase involved in contact‐dependent bidirectional signalling with neighbouring cells	Dasatinib, regorafenib
Histone deacetylase 1 (*HDAC1*)	CP	↑ in A/N/P	Histone deacetylase with regulatory function in transcriptional processes	Romidepsin, vorinostat
Integrin alpha‐V (*ITGAV*)	CP	↑ in Y	Integrin, receptor for a wide array of proteins. CD marker	Antithymocyte immunoglobulin, levothyroxine
Integrin beta‐1 (*ITGB1*)	CP	↑ in Y	Integrin, receptor for a wide array of proteins. CD marker	Antithymocyte immunoglobulin
Mast/stem cell growth factor receptor kit (*KIT*)	CP and CM	↑ in P	Receptor tyrosine kinase, acts as cell‐surface receptor for the cytokine KITLG/SCF. CD marker	Ancestim, imatinib, lenvatinib, pazopanib, regorafenib, ripretinib, sorafenib, sunitinib, tivozanib

Abbreviations: ↑, higher expression; A, SCLC‐A; CD, cluster of differentiation; CM, culture media; CP, cell pellet; FDA: Food and Drug Administration; N, SCLC‐N; P, SCLC‐P; SCLC, small cell lung cancer; Y, SCLC‐Y.

## 4 DISCUSSION

Although clinically SCLC is still regarded as a homogeneous tumour type with NE characteristics, it was described decades ago that besides the ‘classical’ form of SCLC with suspension growth type and NE phenotype, a ‘variant’ form (NE‐low type) of SCLC also exists, mainly forming adherent cell cultures.^62^, ^63^ In addition, tumours lacking NE differentiation (non‐NE) have been also described recently.^6^ In our SCLC cell line panel, 50.0% of the samples grew adherently, whereas the other half semi‐adherently or in suspension. A significant positive relationship between the adherent phenotype and the non‐NE features was observed. Importantly, the proteomic landscape of the examined cell lines also mirrored these phenotypic differences, and proteins associated with several pathways involved in cell adhesion were identified as differentially expressed between adherently or in‐suspension‐growing cell lines, including glycosaminoglycan degradation and endocytosis or gap junction.

Recent profiling studies highlighted that the NE and non‐NE classes of SCLC can be further divided into additional subtypes, namely SCLC‐A and ‐N (NE‐high and NE‐low, respectively) and SCLC‐P and ‐Y (non‐NE types).^6^ Our cell lines were also categorized accordingly using qPCR, resulting in eight, seven, four and seven *ASCL1*+, *NEUROD1*+, *POU2F3*+ and *YAP1*+ cell lines, respectively. In the global proteomic data, these transcription factors showed higher abundances in their respective subtype, with only the elevated level of neurogenic differentiation factor 1 (*NEUROD1*) in SCLC‐N cell lines being not significantly different in all three comparisons.

According to the protein expression of NE and non‐NE markers described by Zhang et al.,^7^ as expected, SCLC‐A and ‐N cell lines showed NE features, whereas those in the SCLC‐P and ‐Y groups were associated with rather non‐NE protein profiles. This is in line with the results of a recent IHC‐based study on human tissue specimens, in which the authors also found that the likelihood of POU2F3 expression in SCLC is quantitatively linked with the level of NE marker expression and SCLC‐P tumours are characterized by the near‐complete absence of NE differentiation.^64^ Therefore, including POU2F3 as a potential additional diagnostic marker might represent an appealing approach for the diagnosis of SCLC tumours that lack or exhibit minimal level of standard NE markers.^64^ Meanwhile, in another study, the same group also showed that the expression of conventional markers linked with NE differentiation is substantially higher in ASCL1‐ and NEUROD1‐defined tumours (vs. ASCL1/NEUROD1‐double‐negative tumours), which as well corresponds with our findings.^12^ Regarding epithelial and mesenchymal traits, a study on SCLC cell lines^65^ outlined that SCLC‐P and ‐Y carry epithelial and mesenchymal attributes, respectively, whereas SCLC‐A and ‐N subtypes have mixed characteristics. Our proteomic study, based on the epithelial and mesenchymal markers established by Kohn et al.,^44^ confirmed these observations. Interestingly, the EMT score was not influenced by the cell lines’ site of origin. Our data also showed that strong NE or EMT characteristics tend to be mutually exclusive.

Importantly, we could categorize our SCLC cell lines into four subgroups based on their comprehensive cellular proteomic profiles, which agreed with the pre‐defined mRNA‐based subtypes (only one cell line was misclassified in proteomics). Of note, the overall high variability of cell media proteins compared to cellular proteins probably interfered with the detection of subtype‐specific signatures in this dataset (M_SD in CP_ = 0.85, M_SD in CM_ = 1.20). Another driver of the proteomic differences was the culture type, affecting both the CP and CM.

To describe the unique traits of SCLC subtypes, we examined the differentially expressed proteins between subtypes and performed a multi‐omic pathway‐level analysis using the expression differences of all quantified proteins (from our dataset) and transcripts (from CCLE transcriptomic data of SCLC cell lines) across the subtypes. Accordingly, we uncovered a list of potential subtype‐specific therapeutic vulnerabilities.

Due to the well‐known regulatory role of *ASCL1* in neural differentiation,^66^ neural precursor cell proliferation and subpallium development–related proteins showed a concordant upregulation in SCLC‐A subtype. Interestingly, proteomic data clearly indicated that the activation of OXPHOS and respiratory chain elements are strongly specific for SCLC‐A. It has been recently described that cell lines not expressing MYC (characteristic for SCLC‐A^6^, ^8^) relies more on oxidative metabolism^67^ suggesting that SCLC‐A tumours might be susceptible to OXPHOS inhibitors.^68^

SCLC‐N cell lines, which predominantly formed suspension cultures in our study, showed a concordant downregulation of cell adhesion pathways. Harvesting the protein‐level data also highlighted the increased activity of DNA replication and transcription and the depletion of cytokine‐mediated signalling in this subtype. Interestingly, a similar trend was described in a study where gene expression differences between SCLC and normal tissue were addressed.^69^

In the epithelial‐like SCLC‐P cell lines, upregulation of the lamellipodium organization pathway was observed, which constitutes a crucial step in EMT leading to increased cell motility and invasive capacities.^70^ In addition, upregulation of neurotrophin signalling foreshadows that poly(adenosine diphosphate‐ribose) polymerase (PARP) inhibitors can be effective therapeutic agents for SCLC‐P as previously proposed,^13^ as prolonged PARP activation was found to contribute to neurotrophin‐induced neuronal death.^71^ Moreover, our data suggests that direct targeting of neurotrophin signalling might be an appropriate treatment option in *POU2F3*‐driven SCLC.^72^

SCLC‐Y cell lines formed a clearly defined subgroup in our SCLC samples. A unique finding in the proteomic data was the downregulation of protein acetylation, chromatin modification (driven by the decreased levels of several histone acetyltransferases) and DNA double‐strand break repair pathways in this subtype. Moreover, we also detected overexpression of the MAPK‐cascade and Rho‐GTPase signalling members in this subtype. Rho is crucial for YAP/TAZ activity^73^; therefore, the upregulation of the latter pathways can be expected in *YAP1+* cell lines. Recently, Caeser et al. reported that in SCLC‐A, the activation of the MAPK pathway rather has inhibitory functions, compared to SCLC‐N/P cell lines. However, no cell lines from the SCLC‐Y subtype were tested by these authors.^74^ In line with the adherent nature of the *YAP1*‐driven cell lines, we also observed that focal adhesion, ECM organization and cell–substrate protein pathways were uniformly upregulated in the SCLC‐Y subgroup, just as peroxisome and endocytosis‐related proteins. Tlemsani et al. described that SCLC‐Y cell lines demonstrate high presenting and native immune predisposition.^65^ Additionally, these cells also have the highest antigen‐presenting machinery scores, thus anticipating that the SCLC‐Y subtype might be sensitive to immune‐checkpoint inhibitors.^65^ In line with this, we also identified a distinctive upregulation of cytokine‐mediated signalling and inflammatory response in this subtype.

Of note, controversies around SCLC‐Y still exists, because comprehensive immunohistochemical and histopathologic analyses of SCLC subtypes in patient samples failed to identify a distinct *YAP1*‐driven subtype.^12^ In contrast, our preclinical proteomic study clearly identifies a unique SCLC‐Y subtype among the examined cell lines. Accordingly, a significant proportion of the 104 proteins with diagnostic relevance was linked to SCLC‐Y. The most promising biomarkers for SCLC‐Y include GPX8, PKD2 and UFO, from which UFO is also potentially detectable in the human blood plasma. This is of clinical importance because the lack of appropriate tissue samples highlights the diagnostic relevance of blood‐based biomarkers. Indeed, so far only a single study attempted to define a panel of subtype‐specific blood‐based biomarkers.^75^ In their study, however, the authors examined the diagnostic relevance of circulating cell‐free DNA, not proteins.^75^ Regarding the potential SCLC‐Y markers identified in our study, gene expression of *GPX8* was previously correlated with YAP1 expression in SCLC cell lines.^76^ Little is known about the role of protein kinase D (PKD) in SCLC, but a previous study has shown that PKD is activated in cell lines H69, H345 and H510 via a protein kinase C‐dependent pathway activation.^77^ The *AXL* gene (encoding the UFO protein) is associated with the mesenchymal phenotype and a potential target for overcoming resistance to epidermal growth factor (EGF) receptor inhibitors.^78^

Our data outlined six proteins that show remarkable differences in abundance across the subtypes and are also FDA‐approved drug targets, namely DDC (overexpressed in SCLC‐A), EPHA2, ITAV and ITB1 (corresponding to genes *EPHA2*, *ITGAV* and *ITGB1*, overexpressed in SCLC‐Y), HDAC1 (downregulated in SCLC‐Y) and KIT (upregulated in SCLC‐P). Cells with high levels of ASCL1 also showed stronger expression of DDC,^79^ whereas EPHA2, a non‐NE marker,^7^ was previously described as upregulated in SCLC‐Y.^65^ Overexpression of integrins in SCLC‐Y contributes to chemotherapy resistance through the suppression of chemotherapy‐induced apoptosis.^80^ In line with this, our group previously reported a positive correlation between YAP1 protein abundance and resistance to chemotherapeutic agents in SCLC cell lines.^9^ An HDAC inhibitor resistance in SCLC‐Y, foreshadowed by HDAC1 downregulation in our study, was also reported recently.^81^ KIT protein is a known SCLC‐P marker.^82^ Importantly, our hypothesis that subtype‐specific overexpression of proteins indicates sensitivity to certain drugs was supported by data from CancerRxGene.^40^ In particular, we confirmed that KIT‐targeting drugs, such as pazopanib, are potentially adequate to target *POU2F3*‐driven SCLCs, whereas *YAP1*‐driven tumours are more resistant to vorinostat (targeting HDAC1).

Among others, the main advantage of cell line–based studies is that one can examine pure populations of homogeneous tumour cells without admixed stromal or inflammatory cells. This is of scientific importance in drug sensitivity assays and subtyping studies such as the current one.^83^, ^84^ Importantly, our proteomic data from cell lines showed a notable overlap with transcriptomic data from SCLC tissue samples in terms of subtype‐specific pathways, suggesting that in vitro investigations of SCLC cell lines are useful in characterizing SCLC tissue subtypes. A comprehensive multi‐omic investigation of SCLC tissue and cell line data by Gay et al. demonstrated the same, namely that SCLCs can be subcategorized both in the presence and absence of tumour microenvironment.^13^

Cell lines, however undoubtedly not ideal models for profiling studies, offer many advantages that complement the use of tumour tissues and animal models for the examination of SCLC.^83^ Nevertheless, some study limitations should be underscored. Although we confirmed several previously described subtype markers, and our results are largely consistent with previous transcriptomic data for subtype‐specific pathways, the total number of cell lines included was relatively small. Additionally, given that all *YAP1*‐driven cell lines had adherent culture type, no subsequent analyses were feasible to investigate subtype and culture type separately. Therefore, the influence of culture type on the distinct characteristics of SCLC‐Y is still unclear. It is also important to note that unique up‐ or downregulation of pathways in a subtype might not translate to dependency or independency from those pathways, as interconnected biological processes and numerous regulatory factors including feedback and feed‐forward loops interfere with such processes. The lack of appropriate treatment‐related data on some cell lines represents another potential study limitation. This is partly because the majority of the examined cell lines were established in the past century, and therefore, the clinicopathological data concerning the tumour they were derived from could not be retrieved even after an in‐depth literature search. Notably, however, neither the NE/mesenchymal characteristics nor the protein expression profile differed significantly according to the presence or absence of chemotherapy (data not shown).

Taken together, our results should primary be considered hypothesis‐generating for future studies, and all finding should be examined in light of the abovementioned limitations. Accordingly, proteomic analyses of larger SCLC cohorts, preferably including fresh tissue samples of patients with homogeneous treatment histories, are needed to validate our findings.

## 5 CONCLUSIONS

To conclude, this is the first large‐scale proteomic study in human SCLC cell lines concerning the newly defined molecular subtypes. We report that SCLC cell lines can be divided into four distinct subtypes by MS‐based proteomics, which accords well with the qPCR‐based classification. Importantly, a distinct *YAP1*‐driven subtype with specific proteomic features could be also distinguished. Comprehensive proteomic profiling of these subtypes has uncovered a list of candidate subtype‐specific therapeutic vulnerabilities for this once enigmatic cancer. Additionally, we also identified several potential IHC‐ and blood‐based biomarkers that might facilitate subtype diagnosis in the future. Altogether, our results might pave the way for future subtype‐specific personalized therapeutic approaches in SCLC.

## CONFLICT OF INTEREST

The authors have no conflicts of interest to declare.

## FUNDING INFORMATION

BS is a recipient of the Semmelweis 250+ Excellence PhD Scholarship (EFOP‐3.6.3‐VEKOP‐16‐2017‐00009). BD, AMS and ZM acknowledge funding from the Hungarian National Research, Development and Innovation Office (KH130356 and KKP126790 to BD; KNN121510 to AMS, 2020‐1.1.6‐JÖVŐ and TKP2021‐EGA‐33 to BD and ZM). BD was also supported by the Austrian Science Fund (FWF I3522, FWF I3977 and I4677). ZM was supported by the UNKP‐20‐3 and UNKP‐21‐3 New National Excellence Program of the Ministry for Innovation and Technology of Hungary, and by the Hungarian Respiratory Society (MPA #2020), PH was supported by the Netherlands X‐omics Initiative (NWO, project 184.034.019). VL is a recipient of the Bolyai Research Scholarship of the Hungarian Academy of Sciences and the UNKP‐19‐4 New National Excellence Program of the Ministry for Innovation and Technology. KS was supported by the Austrian Science Fund (FWF No. T 1062‐B33) and the City of Vienna Fund for Innovative Interdisciplinary Cancer Research. MR acknowledges funding from the Mrs. Berta Kamprad´s Cancer Foundation (FBKS‐2020‐22‐(291)).

## Supporting information

Supporting Information

Supporting Information

Supporting Information

Supporting Information

Supporting Information

## Data Availability

The mass spectrometry proteomics data has been deposited to the ProteomeXchange Consortium via the PRIDE^85^ partner repository with the dataset identifiers PXD029805 and 10.6019/PXD029805 (cell pellet data), PXD029821 and 10.6019/PXD029821 (culture media data). The scripts for the proteomic data analyses can be obtained at: https://github.com/bszeitz/SCLC_proteomics.
