# Supplementary material for: In‐depth proteomic analysis reveals unique subtype‐specific signatures in human small‐cell lung cancer
Source: Clin Transl Med. 2022 Sep 23;12(9):e1060. doi: 10.1002/ctm2.1060 (PMC9506422; doi:10.1002/ctm2.1060)
Supplement: Supplementary file 1 — Supporting Information [file CTM2-12-e1060-s002.docx]

**In-depth proteomic analysis reveals unique subtype-specific signatures in human small cell lung cancer**

**Supplementary tables**

**Supplementary Table 1. The experimental design for the proteomic analysis. (Sheet 1**) Legend. (**Sheet 2**) The experimental design for the cell pellet data. (**Sheet 3**) The experimental design for the cell media data.

**Supplementary Table 2. Differentially expressed proteins between adherent and suspension cell lines, and overrepresentation analysis results.** (**Sheet 1**) Legend. (**Sheet 2**) T-test results for proteins differentially expressed between suspension and adherent cell lines. Overrepresentation analysis results in the cell pellet data (**Sheet 3**) and cell media data (**Sheet 4**) for Gene Ontology, KEGG and Reactome terms using the differentially expressed proteins between suspension and adherent cell lines.

**Supplementary Table 3. Subtype-specific proteins and corresponding overrepresentation analysis results for each subtype.** (**Sheet 1**) Legend. (**Sheet 2**) ANOVA results for proteins showing subtype-specific expression patterns. Overrepresentation analysis results for Gene Ontology, KEGG and Reactome terms using the SCLC-A (**Sheet 3**), -N (**Sheet 4**), -P (**Sheet 5**), and -Y (**Sheet 6**) specific proteins.

**Supplementary Table 4. The pre-ranked GSEA (pGSEA) results before and after summarization.** (**Sheet 1**) Legend. (**Sheet 2**) The detailed pGSEA results for both proteomics and transcriptomics. (**Sheet 3**) The summarized pGSEA results for both proteomics and transcriptomics, showing only subtype-specific processes.

**Supplementary figures**

**
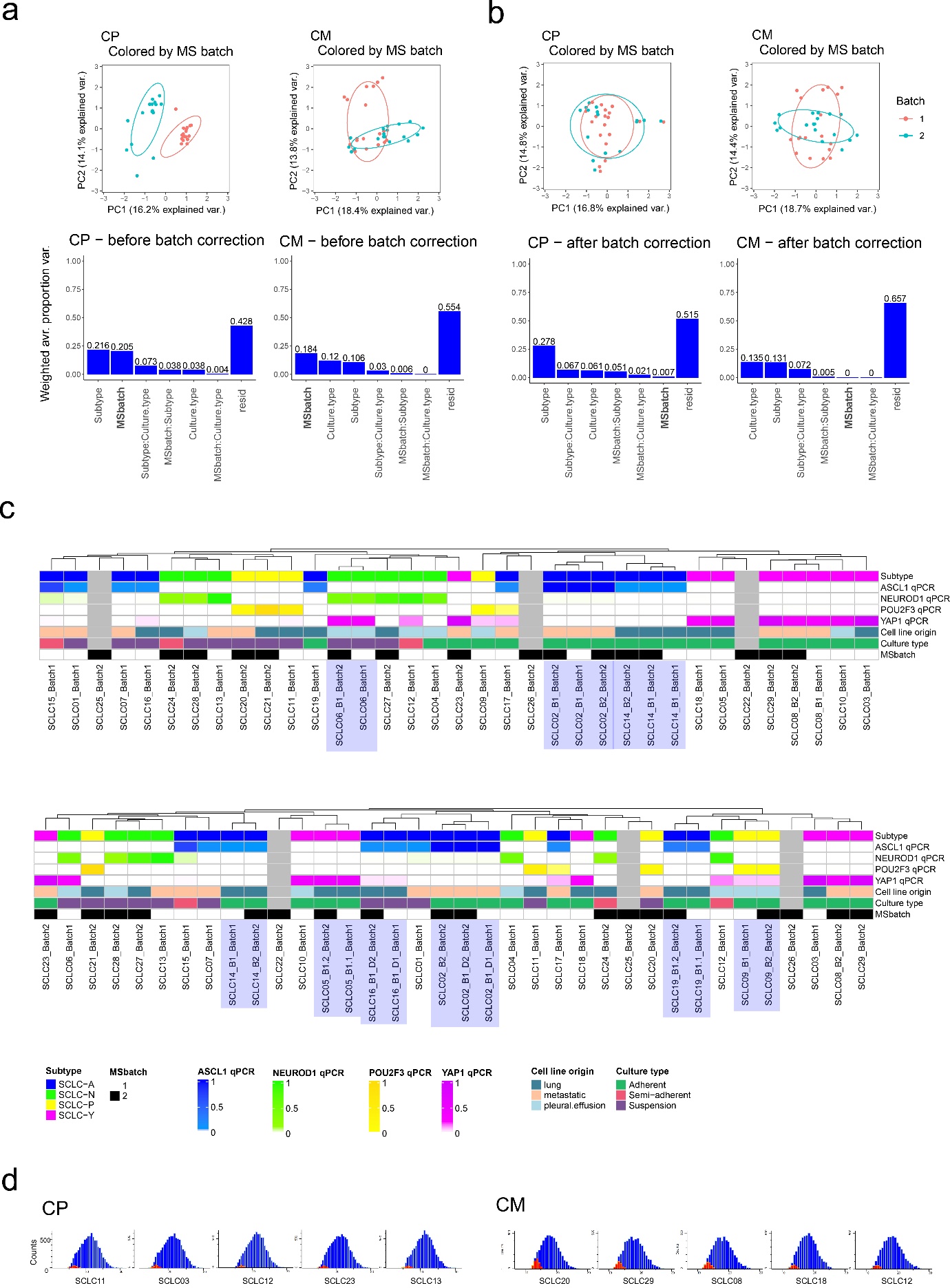
**

**Supplementary Figure 1. Quality control plots of data processing.** (**a**) PCA (top) and PVCA plots (bottom) of the median-normalized data. The residual variation (noted as “resid”) corresponds to the remaining variance in the data set which could not be connected to batch, subtype or culture type effects. (**b**) PCA (top) and PVCA (bottom) plots of the median-normalized and batch-corrected data. The residual variation shows to the remaining biological and technical variance in the data set which could not be connected to batch, subtype or culture type effects. (**c**) Unsupervised hierarchical clustering of the median-normalized and batch-corrected data (settings: complete linkage, Pearson distance). Biological (i.e., repeated cell growth) and technical (i.e., repeated enzymatic digestion for proteomics) replicates displayed high similarity after batch correction. (**d**) Protein intensity histograms for the samples with the top five highest missing value content. Densities of imputed values are highlighted with red.


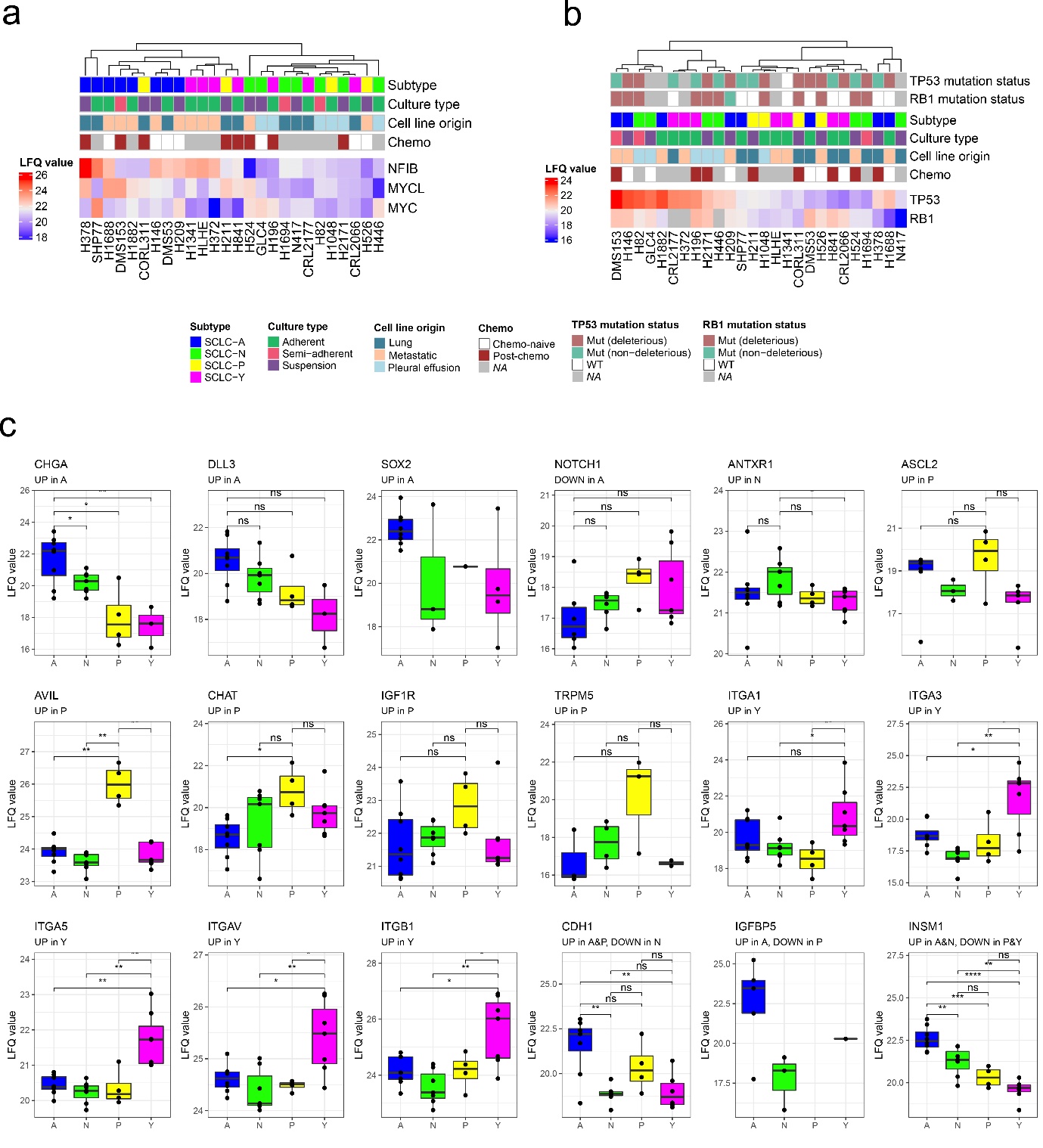


**Supplementary Figure 2. Protein expression profile of selected proteins in our data.** (**a**) LFQ values of NFIB (*NFIB*), MYC (*MYC*) and MYCL (*MYCL*) proteins. (**b**) LFQ values of RB (*RB1*) and P53 (*TP53*) proteins. Missing LFQ values are shown in grey. (**c**) Protein expression profile of well-known subtype markers. The significance of pairwise independent *t*-tests is indicated above the boxplots (ns, p > 0.05; *, p < 0.05; **, p < 0.01; ***, p < 0.001; ****, p < 0.0001).


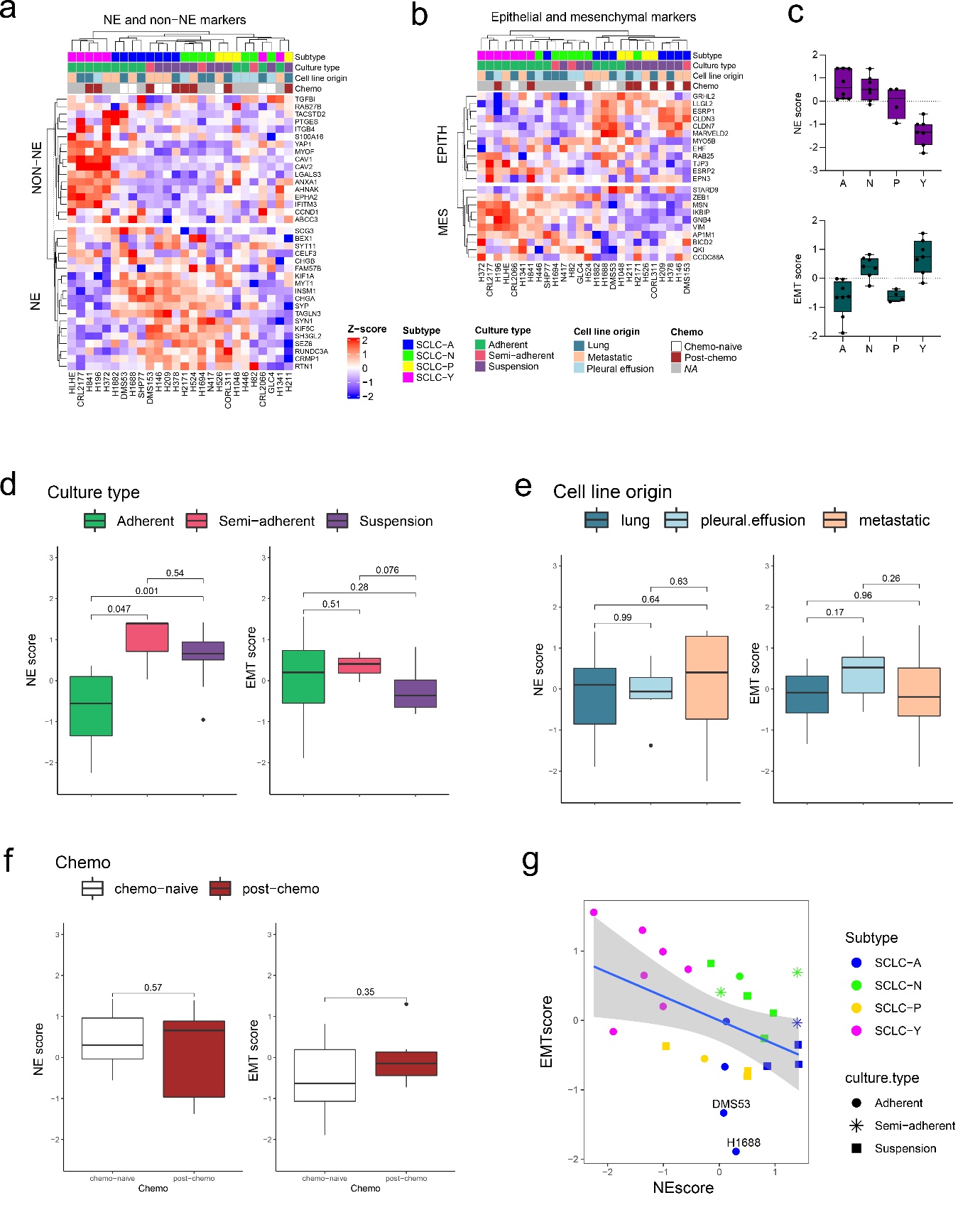


**Supplementary Figure 3. NE and EMT cell line characteristics.** (**a**) Heatmap of NE and non-NE markers. (**b**) Heatmap of epithelial and mesenchymal markers. (**c**) Mean NE (top) and EMT (bottom) scores ±SD within the subtypes. (**d**) Relationship between culture type and NE (left) and EMT (right) scores (*p*-values of pairwise independent t-tests are shown). (**e**) Relationship between cell line origin and NE (left) and EMT (right) scores (*p*-values of pairwise independent t-tests are shown). (**f)** Relationship between chemo-naive and treated cell lines and NE (left) and EMT (right) scores (*p*-values of pairwise independent t-tests are shown). (**g**) Correlation between NE and EMT scores.

**
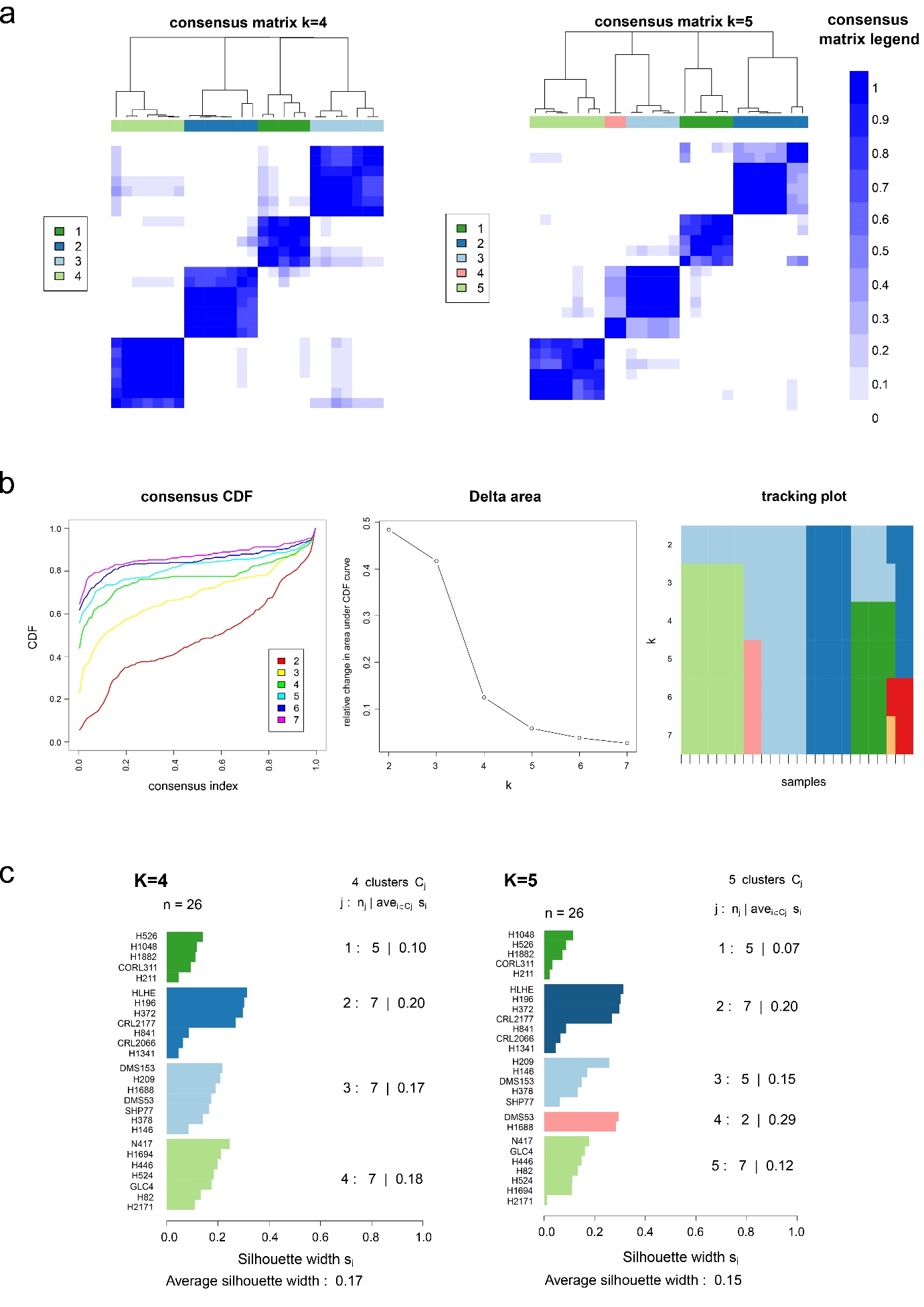
**

**Supplementary Figure 4. Graphical outputs of consensus clustering analysis.** Only proteins with the largest amount of variance (>1.25 SD) were included in the analysis. The clusters were formed using consensus clustering based on 1,000 resampled data sets, exploring the range of 2 to 7 clusters. The Partitioning Around Medoids (PAM) clustering method was used with Pearson distance and complete linkage. (**a**) Visualization of consensus matrices for K=4 and K=5. (**b**) Consensus cumulative distribution function (CDF) and delta area (i.e., change in CDF area) plots for 2-7 clusters, as well as the cluster assignment for each sample for each K (tracking plot). (**c**) Silhouette plots for K=4 and K=5.


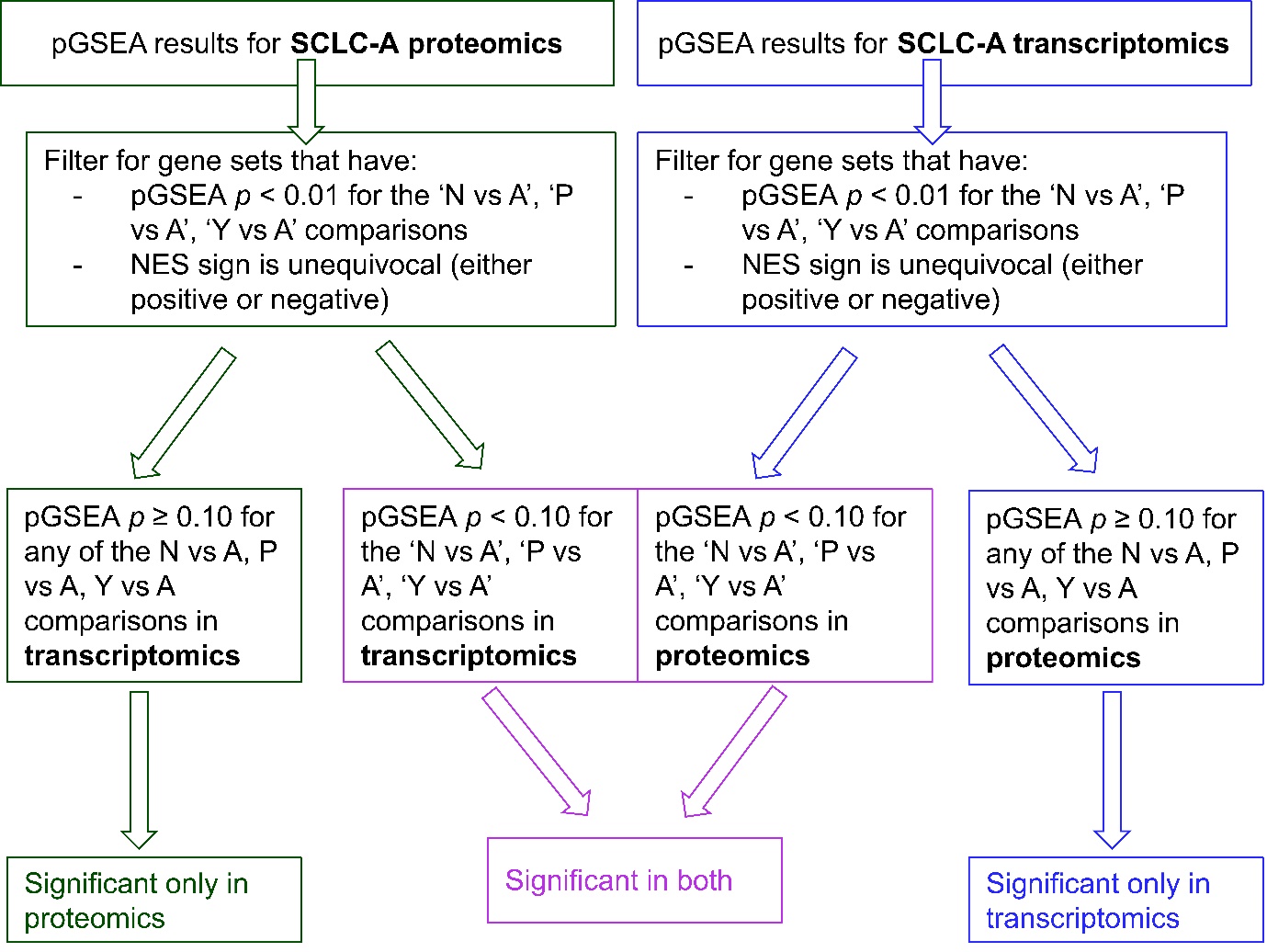


**Supplementary Figure 5. Filtering and comparing pGSEA results between proteomic and transcriptomic datasets.** The scheme above describes the steps used to extract a list of subtype-characteristic gene sets supported by either transcriptomics or proteomics or both datasets. Filtering steps are exemplified on SCLC-A characteristic gene sets.


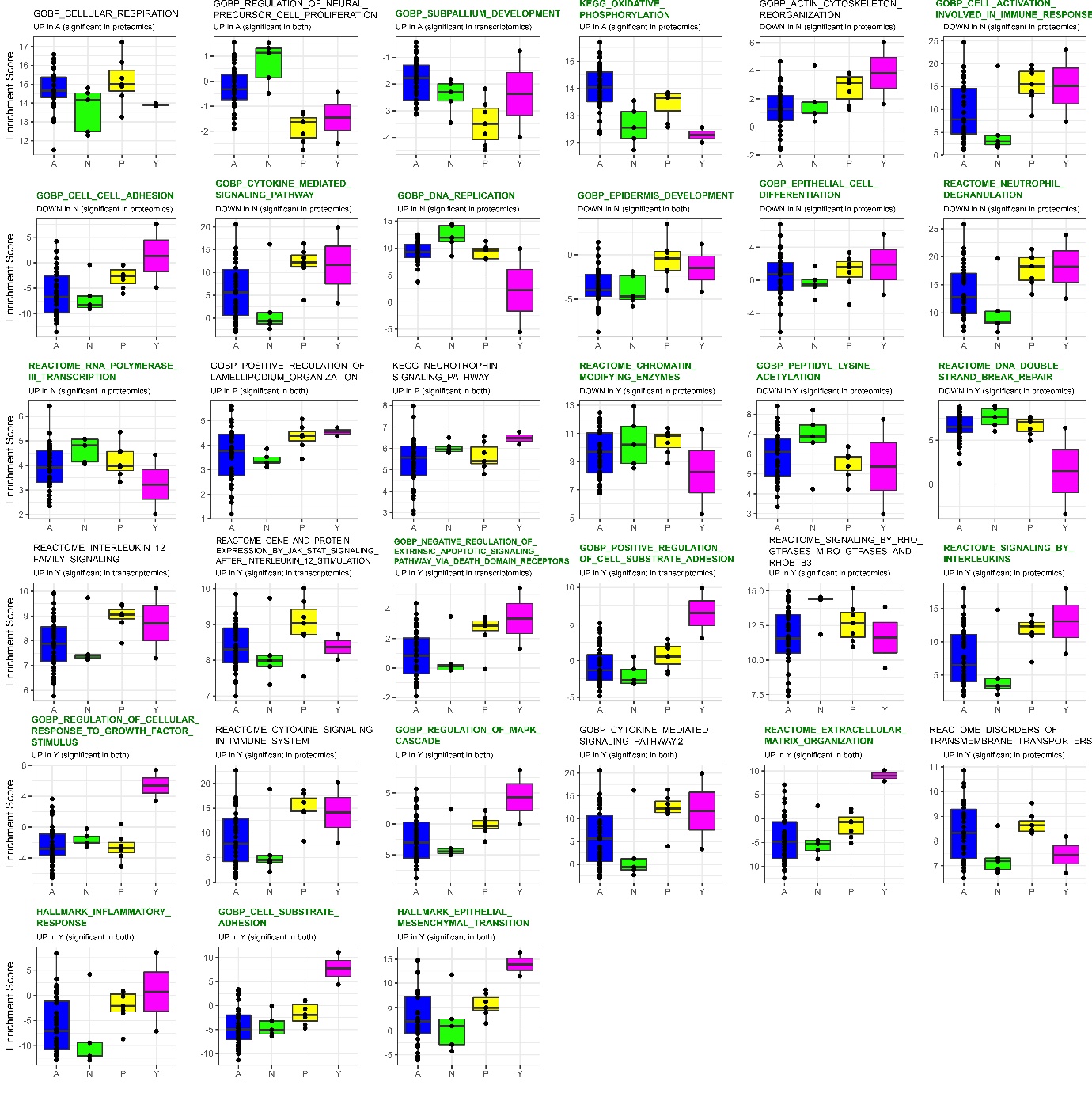


**Supplementary Figure 6. Single-sample GSEA on the SCLC tissue samples (George et al.) using representative gene sets for subtype-specific processes.** Gene set names are highlighted with dark green when the trend for subtype-specificity was observable in the tissue data as well. Y axis shows normalized enrichment scores, and plot titles indicate the results we obtained from the cell line data.


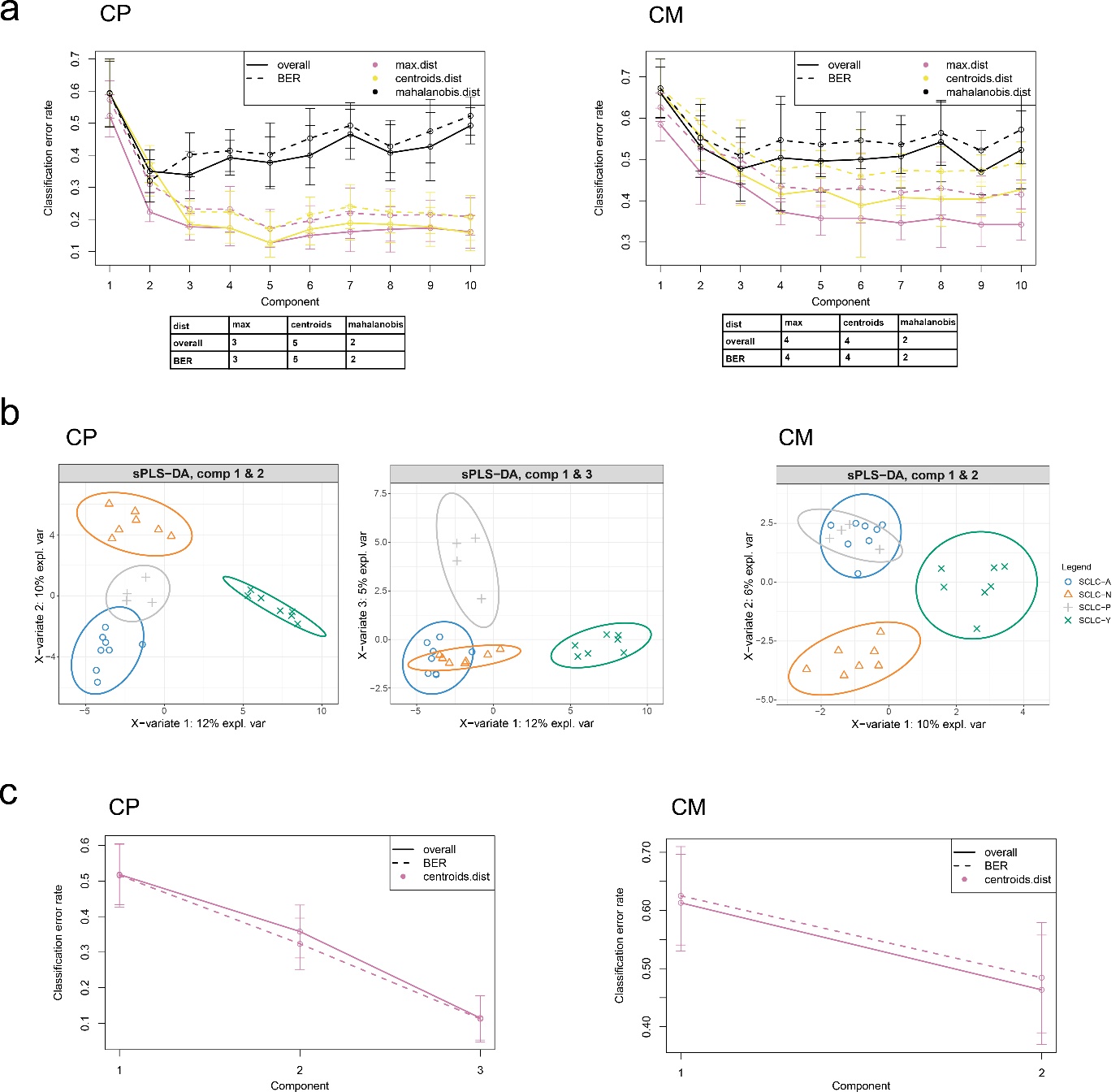


**Supplementary Figure 7. Model tuning for sPLS-DA.** (**a**) The PLS-DA model’s performance plot and number of optimal components using various distance measures (right: CP data, left: CM data). (**b**) The sample plots of the first three and two components based on the final sPLS-DA model for CP and CM respectively. (**c**) Classification performance of the final sPLS-DA model (right: CP data, left: CM data).


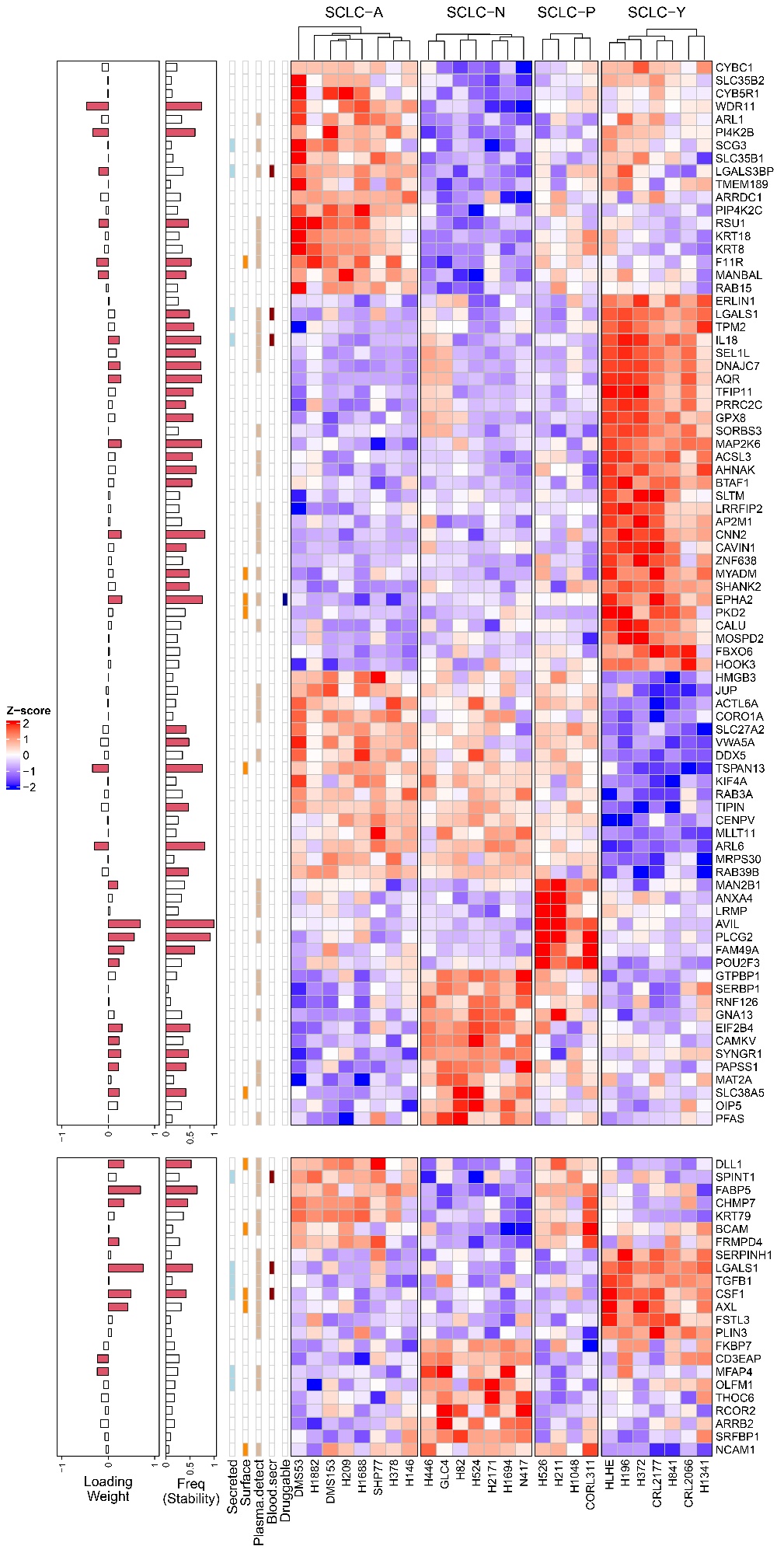


**Supplementary Figure 8. Proteins selected by sPLS-DA to separate the subtypes based on their expression profile.** Top and bottom heatmap show proteins selected in the CP and CM, respectively. Loading weights, frequency values indicating stability and further protein annotations are indicated on the left. Bar plots for the former two row annotations are coloured if the absolute loading weight is larger than 0.2 and if the frequency is larger than 0.4. Gene names of the proteins are indicated on the right side of the heatmap.


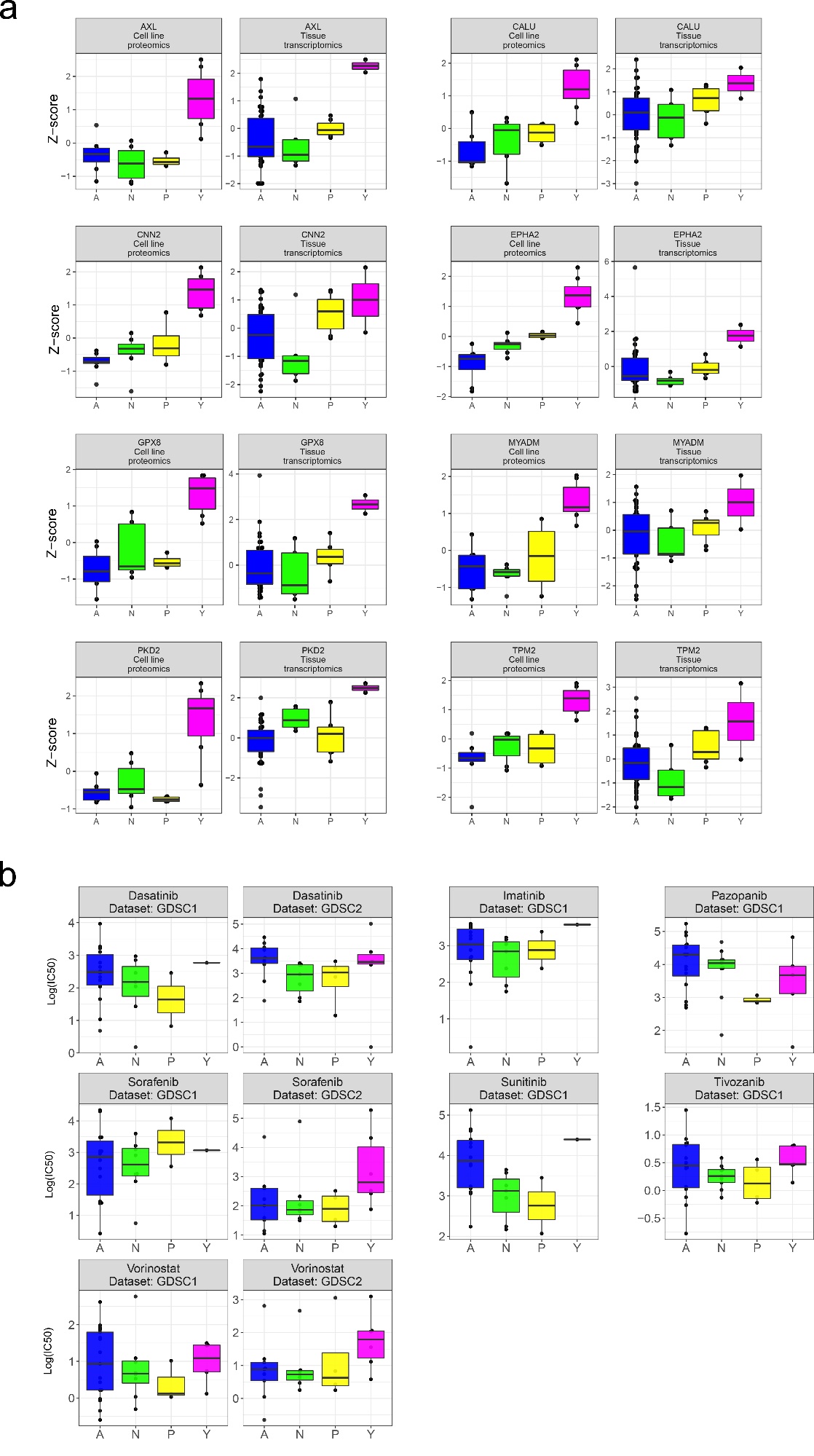


**Supplementary Figure 9. Putative SCLC-Y markers in the George et al. data and differences in drug sensitivity between subtypes.** (**a**) The differential expression of putative SCLC-Y markers between subtypes in our study and in the George et al. data (left and right side of the plots, respectively). Only those genes are shown where the trend was matching with proteomics. (**b**) Differences in drug sensitivity between subtypes, focusing on drugs which target proteins with subtype-specific tendencies.
